# A comparative Proteomics Analysis Identified Differentially Expressed Proteins in Pancreatic Cancer–Associated Stellate Cell Small Extracellular Vesicles

**DOI:** 10.1016/j.mcpro.2022.100438

**Published:** 2022-11-02

**Authors:** Bhaswati Sarcar, Bin Fang, Victoria Izumi, Yury O. Nunez Lopez, Alexandra Tassielli, Richard Pratley, Daniel Jeong, Jennifer B. Permuth, John M. Koomen, Jason B. Fleming, Paul A. Stewart

**Affiliations:** 1 Department of Gastrointestinal Oncology, H. Lee Moffitt Cancer Center and Research Institute, Tampa, Florida, USA; 2 Proteomics and Metabolomics Core Facility, H. Lee Moffitt Cancer Center and Research Institute, Tampa, Florida, USA; 3 Translational Research Institute, Advent Health, Orlando, Florida, USA; 4 Department of Diagnostic and Interventional Radiology, H. Lee Moffitt Cancer Center and Research Institute, Tampa, Florida, USA; 5 Department of Cancer Epidemiology, H. Lee Moffitt Cancer Center and Research Institute, Tampa, Florida, USA; 6 Department of Biostatistics and Bioinformatics, H. Lee Moffitt Cancer Center and Research Institute, Tampa, Florida, USA

**Keywords:** stellate cells, HPSC, HPaStec, PDAC, ultracentrifugation, electron microscopy, small extracellular vesicles, exosome markers, CD63, ESCRT, membrane-associated proteins, liquid chromatography-tandem mass spectrometry, proteomics, exocarta, chromosome segregation 1-like protein, BCA, bicinchoninic acid, BSA, bovine serum albumin, CM, conditioned media, CSE1L, chromosome segregation 1-like, DAPI, 4′,6-diamidino-2-phenylindole, ESCRT, endosomal sorting complex required for transport, EV, extracellular vesicle, FBS, fetal bovine serum, GO: BP, Gene Ontology: Biological Process, GPC1, glypican-1, HPSC, human pancreatic stellate cell, LC-MS/MS, liquid chromatography–tandem mass spectrometry, NTA, nanoparticle tracking analysis, PDAC, pancreatic ductal adenocarcinoma, sEV, small extracellular vesicle, TEM, transmission electron microscopy, UC, ultracentrifugation, α-SMA, α-smooth muscle actin

## Abstract

Human pancreatic stellate cells (HPSCs) are an essential stromal component and mediators of pancreatic ductal adenocarcinoma (PDAC) progression. Small extracellular vesicles (sEVs) are membrane-enclosed nanoparticles involved in cell-to-cell communications and are released from stromal cells within PDAC. A detailed comparison of sEVs from normal pancreatic stellate cells (HPaStec) and from PDAC-associated stellate cells (HPSCs) remains a gap in our current knowledge regarding stellate cells and PDAC. We hypothesized there would be differences in sEVs secretion and protein expression that might contribute to PDAC biology. To test this hypothesis, we isolated sEVs using ultracentrifugation followed by characterization by electron microscopy and Nanoparticle Tracking Analysis. We report here our initial observations. First, HPSC cells derived from PDAC tumors secrete a higher volume of sEVs when compared to normal pancreatic stellate cells (HPaStec). Although our data revealed that both normal and tumor-derived sEVs demonstrated no significant biological effect on cancer cells, we observed efficient uptake of sEVs by both normal and cancer epithelial cells. Additionally, intact membrane-associated proteins on sEVs were essential for efficient uptake. We then compared sEV proteins isolated from HPSCs and HPaStecs cells using liquid chromatography–tandem mass spectrometry. Most of the 1481 protein groups identified were shared with the exosome database, ExoCarta. Eighty-seven protein groups were differentially expressed (selected by 2-fold difference and adjusted *p* value ≤0.05) between HPSC and HPaStec sEVs. Of note, HPSC sEVs contained dramatically more CSE1L (chromosome segregation 1–like protein), a described marker of poor prognosis in patients with pancreatic cancer. Based on our results, we have demonstrated unique populations of sEVs originating from stromal cells with PDAC and suggest that these are significant to cancer biology. Further studies should be undertaken to gain a deeper understanding that could drive novel therapy.

## Highlights

• PDAC-associated stellate cells secrete more sEVs compared to normal stellate cells.
• sEVs reduced α-SMA expressions in normal stellate cells.
• Membrane proteins are essential for stellate cell sEVs uptake by normal and cancer cells.
• LC-MS/MS revealed differentially expressed proteins between HPSC and HPaStec sEVs.
• sEVs could be potentially targeted as cargo vehicles for the safe drug delivery.

## Graphical Abstract

**Figure:**
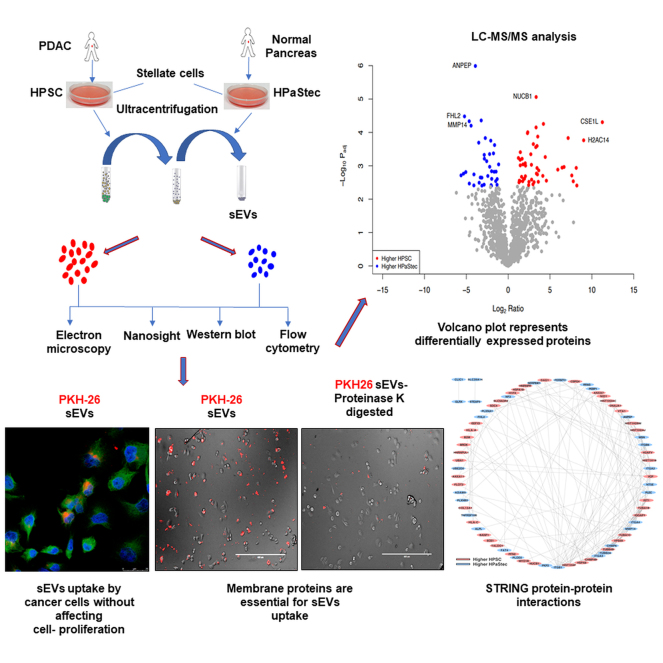

## In Brief

In this article, we report that cancer-associated HPSC cells secreted more sEVs than primary stellate cells (HPaStec) and had no significant growth effects on the cancer cells. Intact membrane-associated proteins may be essential for sufficient uptake of sEVs by both normal and cancer cells. Comparative proteomics analysis between the 2 different sEVs revealed differentially expressed proteins. sEVs could potentially be targeted as a cargo vehicle for the safe delivery of drugs or other biological materials to the cancer cells.

Pancreatic ductal adenocarcinoma (PDAC) exhibits extensive desmoplastic stroma, which is mainly composed of fibroblasts, stellate cells, and immune cells (1). Activated human pancreatic stellate cells (HPSCs) have been reported to impact PDAC development (2, 3). The crosstalk between HPSCs and PDAC and its role in forming desmoplasias are well studied (4, 5, 6). Recent studies have focused on HPSC-moderated tumor progression, survival, and resistance to therapy in PDACs (7, 8, 9). Normal stellate cells are generally quiescent and located in the healthy pancreas (10). Quiescent HPaStecs are engaged in the storage of vitamin A–rich lipid droplets, normal exocrine and endocrine secretion, immunity, and maintenance of normal stromal composition (11). In early PDAC development, normal HPaStecs are activated by various external stimuli and altered molecular pathways. The activated HPaStecs express α-smooth muscle actin (α-SMA), which has been directly correlated with PDAC clinicopathological characteristics and is known as an independent positive prognostic parameter (12). Therefore, exploration of the mechanistic role of activated HPSCs surrounding a PDAC tumor is essential to improve our understanding of intercellular communications involving stromal and tumor cells, which form an intricate network and appear to be an important niche of potential therapeutic targets.

Membrane-enclosed extracellular vesicles (EVs), including small extracellular vesicles (sEVs) and exosomes that were first visualized in rat reticulocytes (13), are significant mediators of cell-to-cell communication and have become a new subject of interest in recent studies (14, 15, 16). sEVs, including exosomes, are secreted from normal and malignant cells into body fluids; they are known functional transporters of proteins, lipids, DNA, and RNAs in intercellular communications (17). sEVs are nanosized particles with diameters ranging from 30 to 200 nm, and they are among the three main types of EVs released to the extracellular environment after the fusion of multivesicular bodies with the plasma membrane. Formation and release of sEVs are mainly characterized by both endosomal sorting complexes required for transport (ESCRT) or ESCRT-dependent and independent manners, although there may be alternative pathways of sEV release (18). sEVs, including endosome-derived exosomes, are mainly enriched with a subpopulation of different tetraspanins, namely, CD9, CD63, and CD81 (19); members of the ESCRT (tumor susceptibility 101 [TSG101], programmed cell death interacting protein or ALIX); and heat shock proteins (HSP60 and HSP90). Multiple studies have focused on sEVs secreted from various cancers and their biological roles in cell-to-cell communication within the tumor microenvironment (20, 21, 22). Studies have also described the mechanisms of exosomal proteins secreted from tumors regulating cancer progression and their role as tumor markers (23). However, it is not well understood how to clinically target exosomal proteins in tumor diagnostic and prognostic evaluations. Additionally, the reported roles of sEVs secreted from the HPSCs surrounding pancreatic tumor cells are controversial. HPSC exosomes were enriched with the proteoglycan, glypican-1 (GPC1), which was suggested as a biomarker to detect PDAC (24); however, another study reported that exosomal GPC1 was unable to differentiate patients with pancreatic cancers from healthy controls (25). HPSC-derived annexin 6A-positive (ANXA6+) EVs containing the annexin A6/LDL receptor-related protein1/thrombospondin1 (ANXA6/LRP1/TSP1) complex promote pancreatic cancer aggressiveness following tumor cell uptake and depletion of ANXA6 in cancer associated fibroblasts impairs tumor metastasis (26). Interestingly, HPSC-derived exosomes provide metabolites and rescue the proliferation of nutrient-deprived MiaPaCa-2 and BxPC3 cells in a KRAS-independent manner (27). Conversely, a recent study revealed that tumor-associated stroma–derived miRNAs are delivered to adjacent PDAC cells *via* exosomes and suppressed tumor cell growth (28). Therefore, a better understanding of the changes in sEVs contents in PDAC-associated HPSCs is needed to gain insight on their role in cancer progression.

Our study included pancreatic stellate cells isolated from primary PDAC tissues (HPSC) and primary stellate cells from normal pancreas (HPaStec). It has been previously reported (29) that HPSCs produce soluble factors that stimulate growth and metastasis of pancreatic cancer. We performed differential ultracentrifugation (UC) on conditioned media (CM) from both HPSCs and HPaStec cells. This process isolated sEVs, including exosomes, which were subjected to morphology tests and physical characterization followed by discovery proteomics with liquid chromatography–tandem mass spectrometry (LC-MS/MS). We describe the exosomes as sEVs throughout the text, according to the current recommendations from the International Society of Extracellular Vesicles (30).

We report here that HPSC cells secreted more sEVs than primary stellate cells (HPaStec). Interestingly, we have shown that intact membrane-associated proteins may be essential for sufficient uptake of sEVs by both normal and cancer cells. Our data revealed that sEVs have no significant biological effects on the cancer cells, but we see this as a benefit since several studies have suggested that sEVs are promising tools for drug delivery across different biological barriers and can be used as next generation therapeutics. Therefore, we performed a detailed comparative proteomics analysis between the two different sEVs, and 87 proteins were differentially expressed. Our results suggested that both sEVs are biologically different from each other and may be utilized as a cargo vehicle for the safe delivery of drugs or other biological materials to pancreatic or other cancer cells.

## Experimental Procedures

### Cell Lines and Reagents

Immortalized HPSCs harvested from primary PDAC tumors were given by Dr Rosa F. Hwang (29) and maintained in RPMI (Invitrogen) medium supplemented with 10% fetal bovine serum (FBS, Invitrogen). Primary HPaStecs isolated from normal pancreases were purchased from ScienCell Research Laboratories and maintained in culture using recommended media (SteCM) at 37 °C in 5% CO_2_. Established cancer and normal pancreas cell lines, such as, Panc1, MiaPaCa2, and HPNE were purchased from ATCC. All experiments were performed using cell lines at less than 10 passages and tested negative for *mycoplasma* contamination using MycoALert Plus *Mycoplasma* Detection Kit (Lonza). The following antibodies were purchased from Santa Cruz Biotechnology: anti-CD63 (sc-5275), anti-ALIX (sc-53540), and anti-TSG 101 (sc-7964). APC anti-human CD63 (#353007) was purchased from BioLegend. The following antibodies were used from Cell Signaling Technology: anti–β tubulin antibody (#2128), anti-Calnexin (#2433), anti-p44/42 MAPK (Erk1/2) (#9102), anti-phospho-p44/42 MAPK (Erk1/2) (#4376), α4 integrin (#4600), β1 integrin (#9699), c-PARP (#5625), c-caspase3 (#9664), and EGFR (D38B1XP) (#4267). Anti-CSE1L/CAS/Exportin-2 (JU34-33) (#NBP2-75451), anti-smooth muscle actin (#MAB1420), and anti–β-actin (#A2228) were purchased from Novus Biologicals, R and D systems, and Sigma-Aldrich, respectively. Gemcitabine hydrochloride and sotrastaurin was purchased from Selleckchem.

### Growth Rate and Cellular Morphology of HPSC and HPaStec Cells

Equal number of HPSC and HPaStec cells (5 × 10^6^) were seeded to assess their growth rate by Trypan blue (Gibco) exclusion method at 24 and 48 h. Cellular morphology of both the cells were captured using Incucyte S3 Live Imager with 10× magnification in-phase channel.

### UC to Isolate sEVs

A classical differential UC protocol (31) was used with minor modifications to isolate sEVs. 5 × 10^6^ HPSC/HPaStec cells were seeded in a T150 cm^2^ flasks (corning) at 50% confluence in each respective medium with 10% exosome-free FBS (Thermo Scientific, #A2720803), and the conditioned medium was harvested when cells were 70% to 80% confluent (48 h). Cell-conditioned media was cleared of cells, cell debris, and large membrane vesicles by sequential centrifugation at 500*g* for 30 min followed by 12,000*g* for an additional 30 min and was then filtered through a 0.22 μM filter unit (Millipore, #SCGP00525). This step was done to maximize quality over quantity to exclude the apoptotic bodies and other debris supported by previous publications (24, 28, 32).To sort by density, sEVs were collected from the cleared supernatants after centrifugation at 100,000*g* for 2 h in SWT32i (Beckman) swinging buckets using a Beckman Coulter ultracentrifuge (Beckman). The sEVs were washed with PBS and repurified by centrifugation at 100,000*g* for 2 h. The pellets were resuspended in 0.22 μM–filtered PBS. Exosomal protein quantity was estimated by Pierce bicinchoninic acid (BCA) protein assay reagent (Thermo Scientific, #23227) and Nanodrop assay (Thermo Scientific), according to the manufacturer’s instructions.

### Electron Microscopy

sEV proteins were quantified by BCA protein assay, and an aliquot of 10 μg protein of the sEVs was fixed with 2.5% glutaraldehyde in Hepes buffer for imaging with transmission electron microscopy (TEM), using a previously described procedure with minor modifications (31). Briefly, 3 μl of fixed sEV suspension was placed on a formvar-coated copper grid (Electron Microscopy Sciences) and allowed to settle for 2 min before adding 3 μl of 2% aqueous uranyl acetate for contrast enhancement. After 30 s, excess liquid was wicked off with filter paper and the grid was allowed to dry overnight before TEM micrograph capture with a JEOL1400 transmission electron microscope (JEOL) equipped with a side mounted Gatan Orius digital camera.

### Western Blot

Cells were lysed in NP40 buffer containing 50 mmol/l Tris [pH 8.0], 150 mmol/l NaCl, 1.0% NP40, and 1× Proteinase Inhibitor Cocktail Set (Thermo Scientific, #1861281). Proteins were estimated using the Pierce BCA protein assay reagent (Thermo Scientific, #23227). Whole-cell protein lysates (40 μg) and sEV proteins (20 μg) were resolved by SDS-PAGE, transferred to Immobilon-P PVDF membranes (Millipore), and incubated overnight in the corresponding primary antibody at 4 °C. Secondary antibodies conjugated to horseradish peroxidase (GE Healthcare) were used, and chemiluminescence (Thermo Fisher Scientific) was used for detection.

### Nanoparticle Tracking Analysis

The size distribution and concentration of particles in the EV preparations were analyzed using dynamic light-scattering technology with a NanoSight NS300 instrument (Malvern Panalytical) and the nanoparticle tracking analysis (NTA) 3.4 software (Malvern Panalytical). The instrument was equipped with a 488 nm blue laser module, flow-cell top plate, integrated temperature control, and a single-syringe pump module. The syringe pump module allowed for flow-controlled detection of moving particles. By tracking the Brownian motion of particles in the suspension, the software calculated the theoretical hydrodynamic diameter of single particles based on the Stokes-Einstein equation. By measuring the light-scattering intensity and the size of individual particles, NTA provided better resolution for heterogeneous mixtures of particles (33). Samples were diluted using cell culture grade water (Corning, #25-005-CI) to produce a particle concentration in the range of 10^7^ to 10^9^ particles/ml (as determined during an initial quick static measurement), which represented approximately 20 to 100 particles/frame (particles in the instrument’s field of view). Final measurements consisted of 5 standard measurements of 1 min of duration each at a controlled temperature of 25 °C and under constant automatic flow (continuous syringe pump speed set to 50 arbitrary units). Camera level for video capture was set to 13 and detection threshold to 5 for all sample measurements.

### Flow Cytometry

Aliquots of 20 μg of sEVs were attached to 10 μl of 4 μm aldehyde/sulfate latex beads (Invitrogen) as described previously (24) with minor modifications. Briefly, to stop the reaction, sEVs-bound beads were treated with 100 mM glycine and 2% bovine serum albumin (BSA) in PBS and were blocked with 10% BSA and incubated with anti-CD63-APC antibody (BioLegend, #353007). Beads alone and beads + sEVs without antibody and beads+sEVs+isotype control (APC igG1, ƙ isotype Ctrl (FC) antibody (BioLegend, #400121) were used as controls to gate the beads with CD63-bound sEVs. The median fluorescence intensity of CD63 (∼100,000 events) was recorded in FACS Canto (BD Biosciences) and results were analyzed using the FlowJo software (BD Biosciences). Experiments were done in three replicates of each conditions.

### Imaging of sEVs by Image Cytometry

Samples were run on an Amnis ImageStream X MKII (ISXII) (Luminex Corporation) with dual charge-coupled device camera system; multiple magnifications (20×, 40×, and 60× objectives); and 405 nm, 488 nm, 561 nm, 642 nm, and 785 nm lasers. The red laser (642 nm, 150 mW) was used to excite CD63-APC–positive sEVs (conjugated to aldehyde/sulfate latex beads) at the power settings of 90 mW, and channel 11 (702/85 nm filter) was used to collect the APC signals. Channels 1 and 9 were used as the brightfield channels, and channel 12 (762/35 nm filter) was used to detect the SSC from the 785 nm laser. All other lasers and channels were disabled during data acquisition. Samples were acquired at low speed and high sensitivity at 60× magnification (7 μm core size, image pixels are 0.33 μm^2^). For all samples, 10 to 20,000 events of CD63-APC+ beads were collected using a data acquisition template created in the INSPIRE acquisition software integrated in ISXII. All data were saved as raw image files as well as FCS files and analyzed using a data analysis template created in IDEAS v6.2 software.

### sEV Uptake

sEVs were labeled with fluorescent lipid dye PKH26 (Sigma) according to the manufacturer’s recommendations with modifications. sEVs were incubated with 2 μM PKH-26, the reaction was stopped using 1% BSA, and extensive washing was done to remove residual lipid dye followed by UC to precipitate labeled sEVs (red). For uptake studies, we have chosen the incubation time and the concentrations of sEVs based on previous publications (34, 35). Subconfluent HPNE, Panc1, and Miapaca2 cells were incubated with PKH-labeled sEVs (20 μg/ml) for 24 h. After incubation, cells were washed twice with 1 M NaCl and twice with PBS to remove cell surface–associated sEVs (34). Cells were fixed with 100% prechilled methanol, permeabilized with 0.2% Triton X-100 (Thermo Scientific), blocked with 10% goat serum (Invitrogen) in PBS, and incubated overnight at 4 °C with the anti-β tubulin antibody (Cell Signaling Technologies). On the next day, secondary antibody (Alexa Fluor 647 anti-rabbit IgG, Invitrogen) was added to the cells prior to counterstaining with 4′,6-diamidino-2-phenylindole (DAPI; Invitrogen) and mounting with antifade. Micrograph images were taken with a Leica SP8 AOBS laser scanning confocal microscope through a 63×/1.4NA Plan Apochromat Oil Immersion Objective Lens (Leica Microsystems CMS GmbH). To excite the samples, 405, 488, and 552 nm laser lines were applied, and tunable emissions were used to minimize crosstalk between fluorochromes. Images were captured with photomultiplier detectors and LAS AF software version 2.6 (Leica Microsystems).

For live cell imaging, HPSC sEVs were digested with proteinase K (Sigma) at the concentration of 40 mAU/mg protein in 37 °C water bath for 1 h followed by heat inactivation of proteinase K in a 60 °C water bath for 20 min. Next, sEVs were labeled with PKH-26 lipid dye as described above. Normal HPNE, Panc1, and Miapaca2 cells were treated with PKH-26 dye only, 20 μg/ml PKH26-labeled HPSC sEVs, or 100 ng of proteinase K–digested PKH26-labeled HPSC sEVs for 6 h. Live imaging was performed on EVOS auto microscope (Leica) at magnification 10×.

### Protein Lysis, Digestion, and Label-Free Proteomics—Sample Preparation

Using equal amounts of protein (10 μg), sEVs were lysed in denaturing buffer containing 8 M urea, 20 mM Hepes (pH 8), 1 mM sodium orthovanadate, 2.5 mM sodium pyrophosphate, and 1 mM β-glycerophosphate. Protease inhibitors were not added because of the use of 8 M urea and to avoid trypsin inhibition during proteolysis. Phosphatase inhibitors were used for future phosphor proteomics experiments. A Bradford assay was carried out to determine the protein concentration. The proteins were reduced with 4.5 mM DTT and alkylated with 10 mM iodoacetamide. Trypsin digestion was carried out at room temperature overnight, and tryptic peptides were then acidified with 1% TFA and desalted with C18 MicroSpin Columns (The Nest Group, Inc) according to the manufacturer’s procedure.

### sEV LC-MS/MS

A nanoflow ultra-high-performance liquid chromatograph (RSLC, Dionex) interfaced with an electrospray benchtop quadrupole-orbitrap mass spectrometer (Q Exactive HF-X, Thermo Fisher Scientific) was used for tandem mass spectrometry peptide sequencing experiments. The sample was first loaded onto a precolumn (100 μm ID × 2 cm in length packed with C18 reversed-phase resin, 5 μm particle size, 100 Å pore size) and washed for 8 min with aqueous 2% acetonitrile and 0.1% formic acid. The trapped peptides were eluted onto the analytical column (C18, 75 μm ID × 50 cm in length, 2 μm particle size, 100 Å pore size, Dionex). The 120 min gradient was programmed as follows: 95% solvent A (aqueous 2% acetonitrile +0.1% formic acid) for 8 min, solvent B (aqueous 90% acetonitrile +0.1% formic acid) from 5% to 38.5% in 90 min, then solvent B from 50% to 90% B in 7 min and held at 90% for 5 min, followed by solvent B from 90% to 5% in 1 min and re-equilibration for 10 min. The flow rate on the analytical column was 300 nl/minute. MS resolution was set at 60 ,000, and MS/MS resolution was set at 15,000 with maximum ion accumulation of 50 ms.

### Data Analysis with Statistical Rationale

MaxQuant (36) (version 1.6.2.10) was used for protein identification and quantification with the Uniprot database (downloaded March 2020; 20,304 total protein entries). Description of the parameters are provided in supplemental Tables S1 and S3. The protease was trypsin, precursor tolerance was 4.5 ppm, and fragment tolerance was 20 ppm. A minimum of one peptide was required for protein identification. At least seven amino acids per peptide were required, and as many as two missed cleavages were allowed. A false discovery rate of 0.01 was used for both peptides and proteins. The match between runs option was selected using a time window of 4 min. N-terminal acetylation and methionine oxidation were both modifications allowed in protein quantification. Membrane annotation was added using Uniprot’s ID mapping tool (https://www.uniprot.org/id-mapping; accessed 2022-06-28) by inputting the first entries of the aforementioned protein groups and then toggling the “Intramembrane” and “Transmembrane” columns in the results.

We used the R programming language (37) (version 4.0.2) with RStudio (38) (version 1.3.1093) to analyze the MaxQuant *proteinGroups*.*txt* output containing 1597 rows of protein groups (see supplemental Table S1, maxquant_params). Filtering out the protein groups that consisted of entirely of reverse entries, entirely nonhuman contaminant entries, or that were identified but not quantified resulted in 1481 protein groups for further analysis. Sample intensities were normalized using iterative rank order normalization (39). Intensities were log_2_-transformed and missing values were left as *NA*. We performed Welch’s t tests to test for differences in the relative expression of protein groups between HPSC and HPaStec samples. *t* test values were adjusted using the Benjamini-Hochberg procedure (40). We defined differentially expressed protein groups as those ≥ 2-fold differences (or ±1 log_2_ ratio of HPSC to HPaStec) with an adjusted *p* value ≤0.05. Ratios were calculated for HPSC over HPaStec, so positive values indicate higher expression in HPSC, whereas negative values indicated lower expression. Pathway enrichment was performed using the Enrichr (41) R package. For protein groups comprised of more than one protein, the first or parent protein of the group was used for enrichment analysis and other comparisons unless noted otherwise. ExoCarta (42) *Protein/mRNA data* (ExoCarta Download - 5 [release date: July 29, 2015]) were downloaded (http://www.exocarta.org/; accessed 2020/09/02). The web-based tool (43) OncoLnc (http://www.oncolnc.org/; accessed 2020/10/28), which provides an interface for multivariate Cox regression, was used to perform a survival analysis on 174 pancreatic adenocarcinomas from The Cancer Genome Data Atlas. Patients were stratified into low and high groups based on median chromosome segregation 1–like protein (CSE1L) expression (43). Significantly, different proteins were queried with the STRING protein–protein interaction database (44). *Homo sapiens* was selected as the organism, and the minimum required interaction score was set to 0.400. The network was exported as a tab separated variable file and imported into Cytoscape version 3.8.2 (45). Circular layout and degree-sorted circular layout options were used to organize the nodes and edges.

### FBS LC-MS/MS

Using a Bradford assay (Pierce Coomassie Plus Bradford Assay 23236), approximately 10 μg of protein from FBS was aliquoted and diluted with 25 mM of ammonium bicarbonate. The sample was subjected to reduction and alkylation of cysteine residues using DTT and iodoacetamide, followed by trypsin at a 1:20 enzyme:protein ratio. After overnight digestion, the sample was acidified with 20% TFA and peptide clean-up was performed using a ZipTip (ZTC18S096 Millipore). Eluted peptides were dried and resuspended in 2% acetonitrile/0.1% formic acid and analyzed by LC-MS/MS in Q-Exactive plus (Thermo Scientific). Data analysis was performed using MaxQuant version 1.6.14 with the Uniprot human database (downloaded March, 2021).

### Fluorescence Microscopy and Western Blot to Detect α-SMA

#### Fluorescence Microscopy

HPaStec cells (1 × 10^4^) were seeded in 8-well chamber slides (Lab Tek II system, Thermo Fisher Scientific), and the next day were left untreated or were treated with HPSC or HPaStec sEVs (20 μg/ml) for 72 h. Cells were fixed with 100% prechilled methanol, permeabilized with 0.2% Triton X-100, blocked with 10% goat serum (Invitrogen) in PBS, and incubated overnight at 4 °C with the anti-SMA antibody (Thermo Scientific,). Cells were incubated with secondary antibody (Alexa Fluor 647 anti-rabbit IgG, Invitrogen) and were counterstained and mounted with antifade-containing DAPI (Invitrogen). Micrograph images were acquired as described above in the sEVs uptake section. Three independent experiments have been done and the immunofluorescence signal of α-SMA in control and treated wells have been quantified using image J software. Western blot: For an additional validation of αSMA expressions, HPaStec cells were left untreated or were treated with HPSC or HPaStec sEVs (20 μg/ml) for 72 h. After 72 h, cells were harvested, lysed, and Western blot was carried out to determine α-SMA expression as described above in the Western blot section.

### Cell Viability Assays Using sEVs

The dose-dependent response of sEVs was evaluated using the CellTiter-Glo assay (Promega). Briefly, either HPNE, Panc1, or Miapaca2 cells (4000 cells/well) were seeded into 96-well plates overnight. The next day, vehicle control (PBS) and increasing doses of sEVs were added to the cells and incubated for 24 and 72 h. For coculture experiments, cancer cells (1000 cells) and stellate cells (1000 cells) were seeded at 1:1 ratio, and the next day, sEVs were added to the cells for 24 h. Cells were incubated at 37 °C with 5% CO_2_ prior to analysis with CellTiter-Glo chemiluminescent reagent (Promega). Data was recorded using Flexstation 3 plate reader (Molecular Devices), and cell viability was normalized to vehicle-treated wells and fit to a sigmoidal dose-response curve using GraphPad Prism 6. All the experiments were performed in triplicate. The data was statistically analyzed using one way ANOVA.

### Cell Viability Assays Using CM and Gemcitabine

Five million cells (HPSC or HPaStec) were seeded in T150 cm^2^ (corning) flasks in a respective media without serum and after 48 h, CM were collected, centrifuged to remove the cellular debris, and concentrated using Amicon ultra centrifugal filtration unit of 100KD cut off (Millipore) (29). Protein contents of the concentrated HPSC and HPaStec CM were determined by Bradford protein assay (Nanodrop) and aliquots were stored at −80 °C until use. Panc1 and Miapaca2 cell lines were seeded in 96-well plates at a density of ∼4000 cells/well and were treated for 48 h to determine the proliferation of Panc1 and Miapaca2 cells in the presence of HPaStec and HPSC CM. Next, for cytotoxicity assay, the cells were treated with gemcitabine (concentration ranging from 0.01–100 μM) alone or in combination with 1 μg/ml HPaStec or HPSC CM (46). To determine gemcitabine-induced cytotoxicity, cell viability in response to varying concentrations of gemcitabine and the corresponding EC50 values were determined using the CellTiter-Glo chemiluminescent assay (Promega). Data analysis was done as described above.

### Live Cell Imaging

The Incucyte (Sartorius Corporation) is a system of exploring *in vitro* biological changes in real-time, automated live-cell imaging, and analysis inside the incubator. Thousands Panc1 and HPaStec cells were seeded in 96-well plates, and the next day, the cells were treated with either vehicle control (PBS) or indicated amount of HPSC-sEVs. Automated live-cell imaging was done for 24 h to explore *in vitro* biological changes in real-time, using Incucyte S3 Live Imager with 10× magnification in-phase channel. Triplicate images were acquired and analyzed from each well.

### Apoptosis and Caspase 3 and 7 Assays

Cells were incubated with HPSC sEVs at 40 μg/ml and 80 μg/ml at various time points following cell lysis and Western blot as described above to probe for apoptosis markers, cleaved PARP, and cleaved caspase 3 (antibodies’ information described in the reagent section). Next, for caspase 3 and 7 and c-PARP assays, HPSC sEVs were incubated with cancer cells at 40 μg/ml and 80 μg/ml for 24 h followed by immunofluorescence to detect the caspase 3 and 7 in the cancer cells using Image-iT Live Red caspase 3 and 7 detection kit (Invitrogen) following the manufacturer’s instructions and to probe for c-PARP respectively. Sotrastaurin was used as positive control for apoptosis. Images were captured at 63× magnification using a Leica SP8 AOBS laser scanning confocal microscope through a 63×/1.4NA Plan Apochromat Oil Immersion Objective Lens (Leica Microsystems CMS GmbH).

### Scratch Assay

The cancer cells were seeded with HPaStec cells in 1:1 ratio into 96-well tissue culture dishes overnight at 37 °C, at a concentration of 2.5 × 10^4^ cells/ml each and cultured in respective medium containing 10% FBS to confluent cell monolayers. The next day, a linear wound was generated in the monolayer with a commercially available tool called wound maker (Essen Bioscience). Cellular debris was removed by washing the cells two times with PBS. HPSC sEVs were added to the cells at a concentration of 40 μg/ml and time lapse imaging was performed by scratch wound assay analysis module in an Incucyte S3 Live imager (Sartorius Corporation) with 4× magnification in-phase channel. Random images were chosen for measurement at 0 h up to 24 h. Experiments were done in quadruplicates and wound confluency and width were plotted in GraphPad Prism.

## Results

### Isolation and Physical Characterization of sEVs Secreted from Stellate Cells

**Fig. 1:**
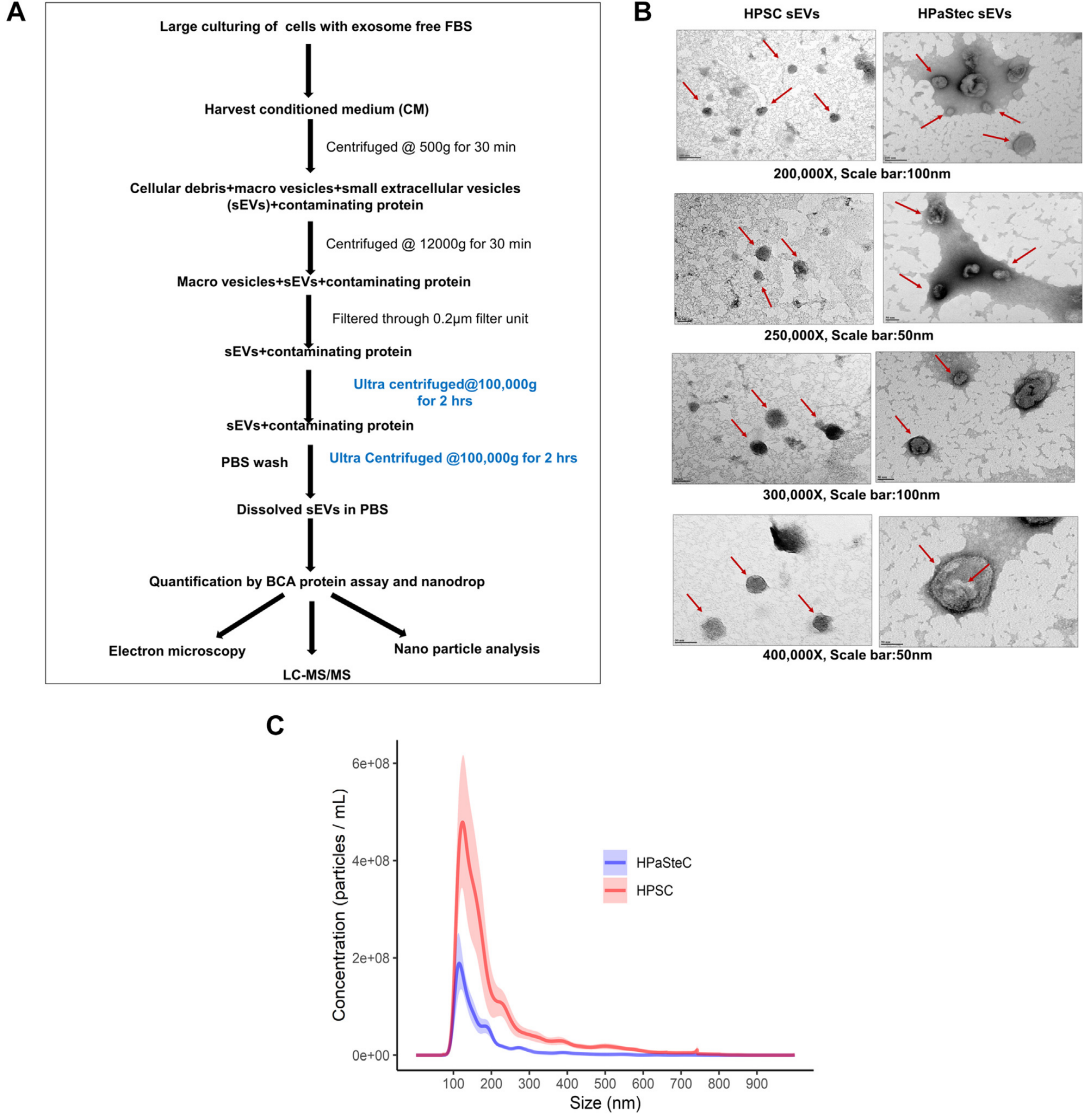
**Isolation and physical characterization of sEVs.***A*, flow chart of sEVs isolation and characterization procedure from stellate cells. sEVs were isolated from the conditioned media of HPSC and HPaStec cells by differential ultracentrifugation and further characterization was done. *B*, electron microscopic characterization of sEVs secreted from stellate cells. Ten micrograms of the sEVs (HPSC and HPastec) were fixed with 4% paraformaldehyde and 1% glutaraldehyde in PBS buffer overnight for imaging in the transmission electron microscope (TEM) using a previously described procedure with minor modification as described in the Experimental Procedures section. Representative electrographs are captured using TEM of HPSC and HPastec sEVs as shown. *Red arrows* are pointing towards the different sizes of sEVs. Image magnifications and scale bars are displayed in the representative images. *C*, NanoSight particle analysis of sEVs show the particle sizes of sEVs in culture supernatants isolated from HPSC and HPaStec cells. The horizontal axis represents the particle size (nm), whereas the vertical axis represents the particle concentration (× 10^8^ particles/ml). The *red bar* represents the SEM. HPSC, human pancreatic stellate cell; sEV, small extracellular vesicle.

HPSCs are like hepatic stellate cells, which are important effector cells in hepatic fibrosis, and stain positive for vimentin, desmin, and α-SMA. Our previous publications described the detailed characterization of immortalized HPSCs isolated from tissues adjacent to PDAC tumors (29, 47, 48, 49, 50). Primary HPaStecs were characterized for the presence of fibroblast marker, α-SMA, as described by the vendor (ScienCell Research Laboratories). In our current study, we have evaluated the morphology and growth rate of both cell lines at 24 and 48 h by seeding equal number of cells. (supplemental Fig. S1, *A*–*C*). There is no significant difference (HPSC vs. HPaStec, unpaired *t* test, *p* = 0.80 and 0.77 at 24 and 48 h) in growth rate between HPSC (1.33 × 10^7^ at 48 h) and HPaStec (1.13 × 10^7^ at 48 h) cells as determined by the cell number (supplemental Fig. S1*A*). For sEV isolation, an equal number of cells were cultured in the respective media using commercially available exosome-free FBS (Invitrogen) until 80% confluency. sEVs were isolated from HPSC and HPaStec-CM using differential UC steps as described previously (31) with minor modifications (Fig. 1*A*). Protein content of the sEVs were then quantified. To analyze the shape and size distributions, 10 μg of sEVs were fixed with glutaraldehyde and stained with uranyl acetate and imaged using TEM. Representative electron micrographs of the clear, round, or ellipsoidal-shaped particles of HPSC and HPaStec sEVs, mostly with a diameter of 50 nm to 130 nm, were visible and were captured using TEM as shown in Figure 1*B* (magnifications ranging from 2,000,00×-400,000×, with a scale bar of 100 or 50 nm, respectively). Although most of the sEVs in both categories fell in the size range of 50 nm to 200 nm, mostly smaller sized HPSC sEVs were captured in the electron micrographs compared to the HPaStec sEVs (Fig. 1*B*).

Next, to confirm the size distribution and determine the concentration of sEVs released from both cell types, we conducted NTA on properly diluted sEV samples (as described in Experimental Procedures) using the NanoSight NS300 (Malvern Panalytical). We have analyzed multiple independent preparations of sEVs using Nanosight NS300 as shown in Table 1. Mean particle diameters of HPSC (six independent preparations) and HPaStec sEVs (five independent preparations) were ranging between 182.2 to 220.9 nm and 154.4 to 191 nm (Table 1 and Fig. 1*C*), respectively (HPaStec vs HPSC, *p* = 0.02). See supplemental Video S1 for additional information. Interestingly, NanoSight quantitation revealed that HPSC cells secrete more sEVs (mean conc.4.75 × 10^10^ particles/ml) compared to HPaStec cells (mean conc.1.35 × 10^10^ particles/ml) from the equal number of cells seeded in an equal volume of CM which is marginally significant (*p* = 0.0569) (Table 1).

**Table 1:** Sizes and concentrations of sEVs determined by NanoSight

Sample	Group	Total concentration	Mean concentration	Mean	Mode	SD	D10	D50	D90
HPaSteC-Prep 1	HPaSteC	6.65E+09	1.35E+10	184.8	125.7	82.5	111.6	154.4	294.9
HPaSteC-Prep 2	HPaSteC	2.02E+10		154.4	111.9	75	103.4	130.8	224.3
HPaSteC-Prep 3	HPaSteC	1.15E+10		191	126.9	111.7	116.1	151.1	319.3
HPaSteC-Prep 4	HPaSteC	2.19E+10		189.1	122.6	99.3	111.9	148.1	335.2
HPaSteC-Prep 5	HPaSteC	7.53E+09		161.8	113.2	72	105.8	141.6	242.8
HPSC-Prep 1	HPSC	7.77E+09	4.75E+10	182.2	123.2	99.5	104.9	145.6	319.3
HPSC-Prep 2	HPSC	4.63E+10		212.2	117.6	131.5	111.9	162.1	403.1
HPSC-Prep 3	HPSC	1.06E+10		198.7	134.7	126.7	112.8	153.4	366.7
HPSC-Prep 4	HPSC	8.7E+10		210.2	138.6	111.3	127	172.9	357
HPSC-Prep 5	HPSC	5.09E+10		191.9	114.1	93.7	107.8	154.7	342.9
HPSC-Prep 6	HPSC	8.25E+10		220.9	119.8	141.1	111	155.9	437.7
**HPaSteC *versus* HPSC***t* test		0.0569		0.020^a^	0.405	0.025^a^	0.503	0.057	0.011^a^

SD: standard deviation of mean particle size. D10: 10 percentile diameter. Ten percent of the particles in the sample are smaller than the D10 diameter. D50: 50 percentile diameter. Fifty percent of the particles in the sample are smaller than the D50 diameter. D90: 90 percentile diameter. Ninety percent of the particles in the sample are smaller than the D90 diameter.

a indicates differentially expressed between HPSC and HPaStec.

**Fig. 2:**
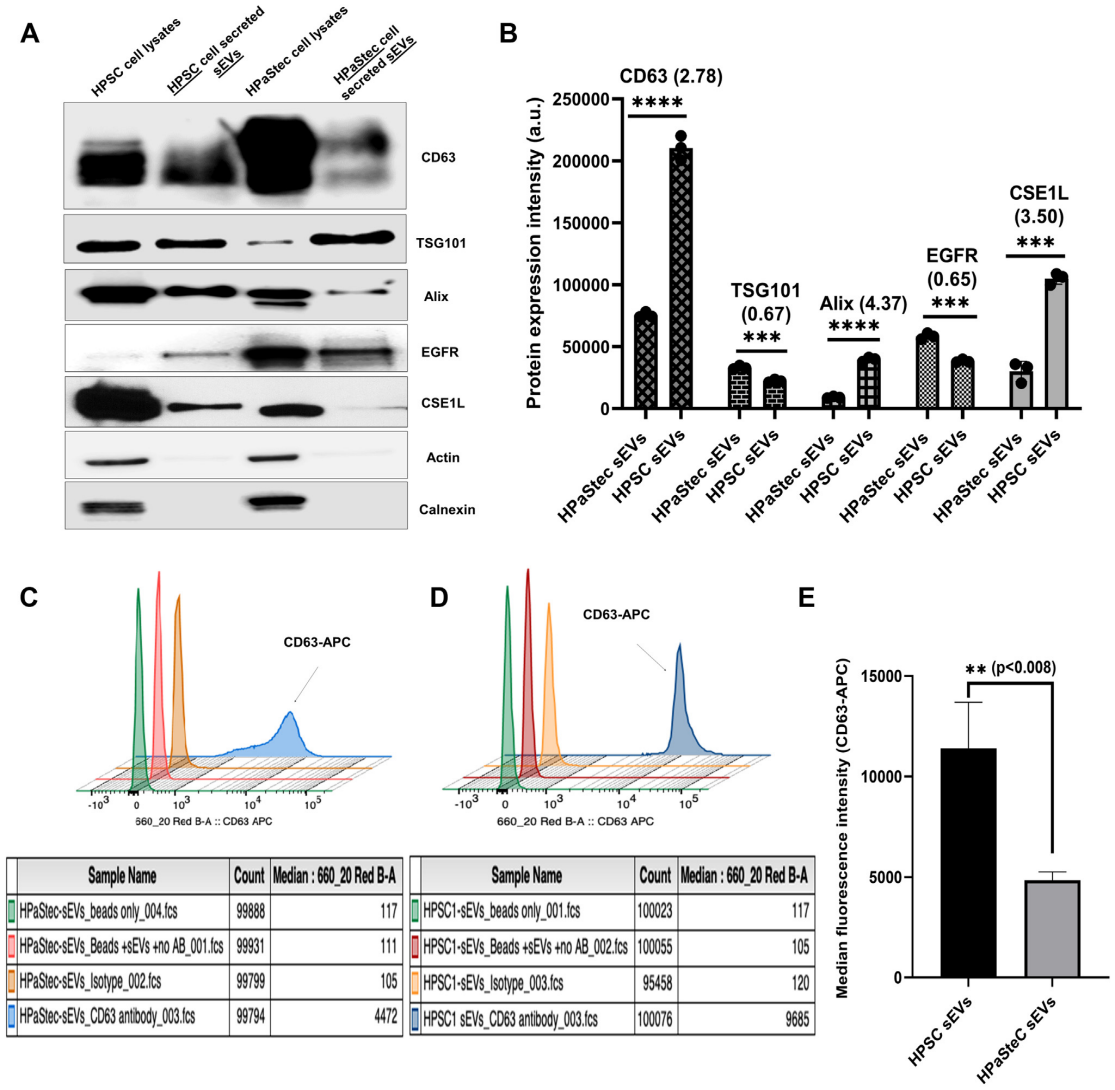
**Characterization and quantification of sEV and exosomal markers in sEVs secreted from stellate cells**. *A*, Western blot analysis was performed on HPSC and HPaStec sEVs with the parental cells to examine exosomal markers’ (Alix, TSG101, CD63, EGFR, and CSE1L) expression and any endoplasmic reticulum (Calnexin) contamination in sEVs using 20 μg of total protein loaded in each lane. *B*, protein expression intensities (a.u.) were compared between HPaStec sEVs and HPSC sEVs using image J software and the fold increase were indicated on the graph (HPSC sEVs vs. HPaStec sEVs, CD63: 2.78 (∗∗∗∗*p* < 0.0001); TSG101:0.67 (∗∗∗*p* < 0.0005); Alix: 4.37 (∗∗∗∗*p* < 0.0001); EGFR: 0.65 (∗∗∗*p* < 0.0002); CSE1L: 3.50 (∗∗∗*p* < 0.0002)). *C* and *D*, histogram of median fluorescence intensity of CD63-APC (Y-axis) (*blue*) of HPaStec (*C*) and HPSC (*D*) sEVs on beads of a representative experiment. Aliquots (20 μg) of each type of sEVs were incubated with aldehyde latex beads and incubated with beads alone (*green*) and beads + sEVs (*red*) without antibody and beads+sEVs+isotype control (*orange*) (APC igG1, ƙ isotype Ctrl (FC) antibody were used as controls to gate the beads with CD63-bound sEVs and with CD63-APC antibody (blue) as described in Experimental Procedures section and analyzed in BD FACS Canto (BD Biosciences). *E*, GraphPad analysis showed the difference in median fluorescence intensity of CD63 between HPSC SEVs and HPaStec sEVs of three independent experiments by one-way Anova (∗∗*p* < 0.008). HPSC, human pancreatic stellate cell; sEV, small extracellular vesicle.

To further characterize differences in these sEVs, Western blot analyses were performed to detect expression of established exosomal surface marker proteins (Alix, CD63, TSG101, EGFR, and CSE1L) and any cytoskeletal (Actin) or endoplasmic reticulum (Calnexin) contaminations in the sEVs. These surface markers were present in sEVs, and the extracts were free of cytoskeletal/endoplasmic reticulum contamination (Fig. 2*A*). We have quantified the Western blot analyses using ImageJ bundled with Java 1.8.0_172 software (Fig. 2*B*). HPSC sEVs were significantly more enriched with most of the exosomal markers compared to HPaStec sEVs (Fig. 2*B*). Next, flow cytometry analyses were performed in HPaStec (Fig. 2*C*) and HPSC(Fig. 2*D*)-sEV–bound beads to analyze the expression of CD63, one of the tetraspanin exosomal markers (51, 52). The representative histogram with proper isotype control in Figure 2, *C*, and *D* and mean bar graph in Figure 2*E* (three independent experiments) showed that the median fluorescence intensity of CD63 was significantly 2-fold higher (*p* < 0.008) in HPSC than HPaStec sEVs. ImageStream analysis revealed that staining of APC-CD63–bound HPSC sEVs was brighter than the HPaStec sEVs (supplemental Fig. S2, *A*–*D*).

### Coculturing of sEVs with Normal HPaStec Cells Modulates α-SMA Expression

**Fig. 3:**
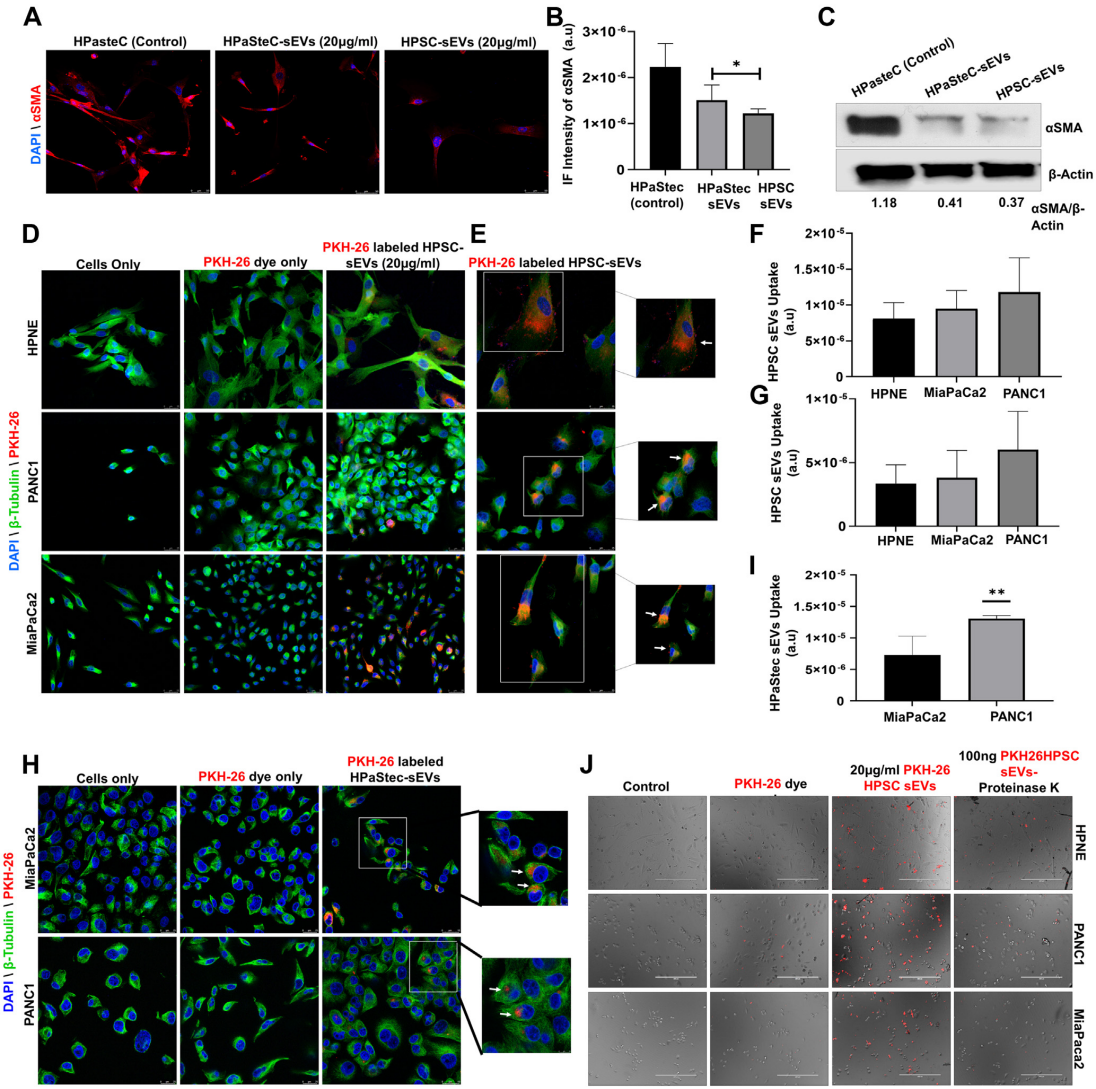
**Effect of sEVs on normal stellate cells and uptake of PKH-26–labeled sEVs by normal epithelial and cancer cells:***A*, HPaStec cells were *left* untreated or treated with either HPSC or HPaStec sEVs (20 μg/ml) for 72 h. Cells were fixed and stained with anti-α-SMA antibody (*red*) and DAPI (*blue*, DNA dye) as described in the Experimental Procedures. Representative 63× images (scale bar represents 50 μm) using confocal microscopy showed significantly decreased staining of α-SMA in HPaStec cells treated with both HPastec sEVs and HPSC sEVs. *B*, representative immunofluorescence intensities (α-SMA) of control and treated wells were quantified using image J software, and statistical analysis was done using one way ANOVA (∗*p* < 0.03). *C*, HPaStec cells were treated with 20 μg/ml HPSC-sEVs or HPaStec sEVs for 72 h, and Western blot analysis was performed using anti-α-SMA antibody and actin is used as a loading control. Percentage signal intensities of αSMA were normalized against actin as indicated using image J software. *D* and *H*, immunofluorescence of fixed cells to demonstrate the uptake of PKH-26–labeled HPSC/HPaStec sEVs. Normal HPNE, Panc1, and Miapaca2 cells were treated either with PKH-26 dye (*red*) only or with 20 μg/ml PKH26-labeled HPSC sEVs for 24 h. Following incubation with anti-tubulin antibody to stain the cytoplasmic membrane (*green*), imaging was done using confocal microscopy (63× Magnification, scale bar represents 25 μm for HPSC sEVs and scale bar represents 10 μm for HPastec sEVs). *E*, the images at 1890× magnification visualize the cellular localization of PKH-26–labeled HPSC sEVs in HPNE, Panc1, and Miapaca2 cells. *White arrows* show the localization of sEVs to the cell membranes. *F*, *G*, and *I*, uptake of both PKH-26–labeled sEVs by cancer cells were quantified using image J software and statistical analysis was done using unpaired *t* test (HPaStec sEVs uptake in Panc1 cells, (∗∗*p* < 0.02). *J*, live cell imaging to demonstrate preferential sEV uptake. Normal HPNE, Panc1, and Miapaca2 cells were treated either with PKH-26 dye (*red*) only or with 20 μg/ml PKH26–labeled HPSC sEVs or with proteinase K digested 100 ng (A260/A280 = 1.84) PKH26-labeled HPSC sEVs for 6 h sEV delivery to normal and cancer cells was higher when undigested sEVs were added to the cells. Live imaging was performed on EVOS auto microscope (Leica) at 10× magnification (scale bar represents 400 μm). DAPI, 4′,6-diamidino-2-phenylindole; HPSC, human pancreatic stellate cell; sEV, small extracellular vesicle; α-SMA, α-smooth muscle actin.

To explore the activation of normal stellate cells in the presence of HPSC and HPaStec sEVs, both sEVs were cocultured with normal HPaStecs at a concentration of 20 μg/ml for 72 h followed by α-SMA staining (activation marker for stellate cells). Interestingly, based on our data, by sEVs treatment, both types of EVs reduced the α-SMA expression significantly (*p* < 0.03) relative to untreated control as detected by immunofluorescence and Western blot (Fig. 3, *A*–*C*).

### Uptake of sEVs by Normal Epithelial Cells and PDAC Cell Lines

Next, to study the uptake of both HPSC and HPaStec sEVs by normal and cancerous cell lines, we labeled the sEVs with a red fluorescent dye (PKH-26) that has long aliphatic tails that are incorporated into the lipid membrane. Next, 20 μg/ml of PKH-26–labeled sEVs were incubated with normal epithelial cells, HPNE, and PDAC cell lines, Miapaca2 and Panc1 for 24 h. The cells were fixed with methanol and to demonstrate the localization of sEVs, cells were stained with β-tubulin, which represents the cytoskeletal (green) structure of the individual cells and DAPI (blue) counterstained the nucleus. Immunofluorescence imaging using a confocal microscope determined the overall uptake of PKH-26–labeled HPSC sEVs (red) in normal and pancreatic cancer cells (Fig. 3, *D* and *E*) and uptake of HPaStec sEVs in panc1 and Miapaca2 cells (Fig. 3*H*). Higher magnifications (1890X) of the images revealed that most of the sEVs were accumulated on the cellular membranes of cells (Fig. 3, *E* and *H*). There are no significant differences in HPSC sEVs uptake between the cell lines (Fig. 3, *F* and *G*). HPaStec sEVs uptake by Panc1 cells are significantly higher than Miapaca2 cells. (Fig. 3*I*).

### Membrane Proteins on the sEVs may be Essential for Better Uptake of sEVs in Normal and Cancer Cell Lines

To determine whether HPSC-derived sEVs enriched with membrane proteins are essential for better uptake of sEVs by normal or cancerous cells, undigested PKH-27–labeled HPSC sEVs (20 μg/ml) or 100 ng of proteinase K–digested PKH-26–labeled HPSC sEVs were treated to HPNE, Panc1, and Miapaca2 cells for 6 h. Uptake of sEVs by the live cells was visualized *via* live imaging with the EVOS FL Auto Microscope (Thermo Scientific) (Fig. 3*J*). Digestion of exosomal surface proteins with proteinase K significantly reduced the entry of sEVs into HPNE, PANC1, and Miapaca2 cells (Fig. 3*J*) than the higher levels of uptake of undigested sEVs by normal (HPNE) and cancer cells (Panc1 and Miapaca2).

### sEVs Have no Significant Effect on Cellular Proliferation of Cancer Cells

Next, to evaluate the effects of HPSC sEVs and HPaStec sEVs on the proliferation of normal epithelial cells and pancreatic cancer cells, HPNE and Panc1 or Miapaca2 cells were coincubated with indicated amount of either HPSC sEVs or HPaStec sEVs for 24 and 72 h. We could not find any significant effects of either sEVs on proliferation of normal epithelial or cancer cells, as shown in supplemental Fig. S3, *A*–*E*. Next, to get better insight on the effect of stellate cells and sEVs on the proliferation of cancer cells in the presence of normal stellate cells, primary HPaStec and PDAC-associated HPSC cells were cocultured with either Panc1 or Miapaca2 cells for 24 h either in the presence or absence of HPSC/HPaStec sEVs. Interestingly, there were no significant changes in cellular proliferation observed upon addition of HPSC or HPaStec sEVs to the coculture system (supplemental Fig. S3, *F* and *G*). We also demonstrated that there were no changes in cellular morphology of both primary stellate cells (HPaStec) and Panc1 cells in the presence or absence of HPSC sEVs (supplemental Fig. S3*H*).

We further examined the cellular apoptosis and migration of Panc1 and Miapaca2 cells in the presence of HPSC sEVs. Interestingly, HPSC sEVs did not significantly induce any c-Caspase 3/7 or c-PARP to the cancer cells as shown by Western blots (supplemental Fig. S4, *A* and *B*) and immunofluorescence (supplemental Fig. S4, *C* and *D*). Cellular migration of Panc1 and HPaStec cells in the presence of HPSC sEVs also remained unchanged (supplemental Fig. S4, *E*–*G*).

Next, to examine whether soluble part of the CM but not EV most likely has an effect on cancer cells, HPSC/HPaStec concentrated CM were cocultured with Panc1 and Miapaca2 cells with indicated time as described previously (29) (supplemental Fig. S5, *A* and *B*). Coculture of Panc1 and Miapaca2 cells with HPSC stellate cells CM resulted in significant increase (*p* < 0.0001) of the growth of Panc1 cells only (supplemental Fig. S5*A*). Effects of either CMs in Miapaca2 cells remained unchanged (supplemental Fig. S5*B*). According to the previous report, pancreatic stellate cells are capable of provoking chemoresistance of tumor cells (53). Therefore, we treated the cancer cells with chemotherapeutic drug gemcitabine, the standard of care for the pancreatic cancer (54), alone or in combination with HPSC/HPaStec CMs. However, according to our findings, HPSC/HPaStec CMs are unable to induce significant chemoresistance (no significant changes in the EC50 values. (supplemental Fig. S5, *C* and *D*).

### Proteomics Identified Differences Between Normal and Cancer-Associated Human Pancreatic Stellate Cell sEVs

**Fig. 4:**
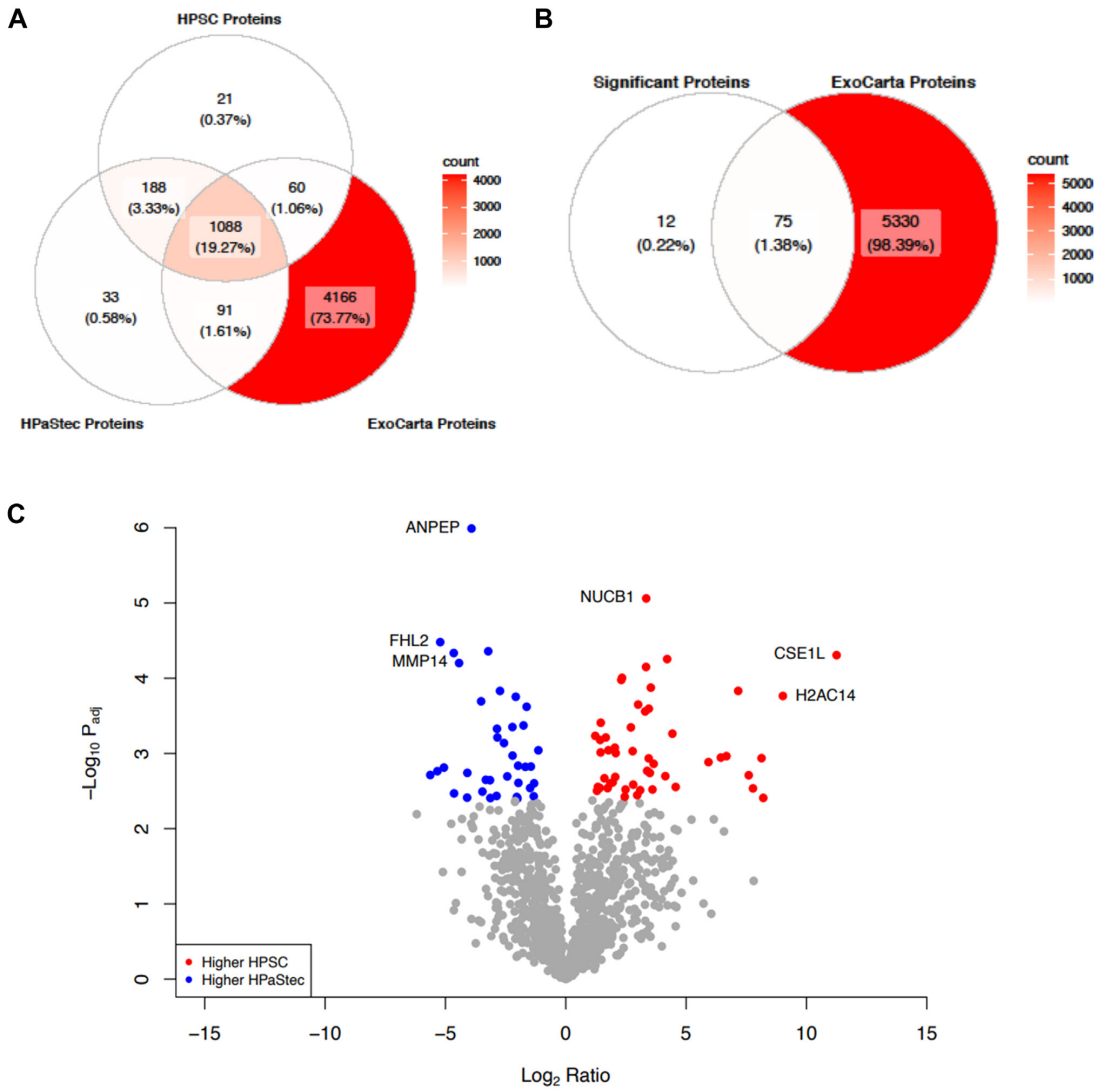
**Venn diagrams and Volcano plot of differential protein expression levels of HPSC and HPastec sEVs**: *A*, Venn diagram of exosomal proteins found in Exocarta. Overlap of identified protein groups by sample type and ExoCarta proteins. A large percentage of identified protein groups was also found in ExoCarta, which was consistent with sEV enrichment. *B*, significant proteins and overlap of the differentially expressed protein groups and ExoCarta. *C*, volcano plot of the 1481 proteins from HPSC and HPastec sEVs. The 50 protein groups with at least two-fold higher expression (or 1 log_2_ ratio) with an adjusted *p*-value ≤ 0.05 are colored *red* and 37 protein groups with two-fold lower expression (or -1 log_2_ ratio) with adjusted *p*-value ≤ 0.05 are colored *green*. Ratios are given relative to HPSC. CSE1L was noticeably higher (log_2_ ratio 11.25) in HPSC sEVs. HPSC, human pancreatic stellate cell; sEV, small extracellular vesicle.

PDAC-associated HPSC cells secrete more exosomal sEVs than HPaStec and intact membrane protein–containing sEVs are better taken up by cancer cells. Therefore, to test whether the membrane protein expression levels were different in sEVs of HPSC and HPaStec cells, both the cells were grown in biological triplicates. sEVs were harvested and analyzed using LC-MS/MS proteomics. After processing with MaxQuant for quantification (36) and quality control, we identified 1481 protein groups for further analysis. Of these 1481 protein groups, 355 proteins had transmembrane annotation and 9 had intramembrane annotation in Uniprot (supplemental Table S2). We compared our findings to the supplemental data available that have also compared vesicles from tumor-associated and normal stellate cells and in total, we have 76% protein identification overlap with findings from Servage *et al* (supplemental Tables S2, S3, supplemental Fig. S6) (55). Data were log_2_ transformed and normalized using iterative rank order normalization (39) (supplemental Fig. S7, *A* and *B*). Principal component analysis showed that HPSC and HPaStec sEVs separated in principal component 1 (supplemental Fig. S7*C*). Consistent with exosome enrichment, 1239 protein groups were listed in the ExoCarta database (42) and 1088 ExoCarta proteins were identified in exosomes from both cell types (Fig. 4*A* and supplemental Table S3). Sixty or ninety-one ExoCarta proteins were exclusively identified in HPSC or HPaStec sEVs, respectively (Fig. 4*A*).

During our sEVs isolation we did not use serum-free media, so we washed the pellets with PBS after sEVs precipitation. Since there is a minor risk of FBS contamination, we independently ran an FBS aliquot through LC-MS/MS, searched with the human Uniprot database, and intersected with our results. Overlap with our findings and FBS was small (2% of identifications or 30 proteins), as seen in supplemental Table S3 and supplemental Fig. S8.

We performed Welch’s t tests and adjusted the *p* values using Benjamini-Hochberg (40) methodology (supplemental Fig. S7) to test for differences in protein content between HPSC and HPaStec sEVs. We identified 87 protein groups that were differentially expressed (±1 log_2_ ratio of HPSC to HPaStec and adjusted *p* value ≤0.05; Figure 4*B* and volcano plot in Fig. 4*C* and supplemental Table S3). Among the top 10 proteins elevated in HPSC sEVs (Tables 2 and S3), CSE1L, also known as cellular apoptosis susceptibility protein (CAS) and Exportin-2 (XPO2), was dramatically higher (log_2_ ratio = 11.25, *P*_adj_ = 0.009). Other notable results included that HPSC sEVs were enriched with several DNA-binding histones (Table 2 and supplemental Table S3), such as histones H2A type 1, core histone macro-H2A.1, H2A.V, H2B type 1, H1.5, and histone H4. Other than histones, chromatin regulatory barrier to auto integrin factors (BANF1) and BRO1 domain-containing protein (BROX) were highly enriched in HPSC sEVs (Table 2 and supplemental Table S3). There were significantly lower amounts of an aminopeptidase, ANPEP, and a matrix metalloprotease, MMP14, (supplemental Table S3). Notably, except for annexins 2 and 3, HPSC sEVs were enriched with annexins 1, 4, 5, 6, 7, and 11 compared to HPaStec sEVs (Table 3). Several integrins were elevated and differentially expressed in HPaStec sEVs: integrin alpha-2 (ITGA2), integrin alpha-4 (ITGA4), integrin 1 (ITGB1), integrin beta-6 (ITGB6), and integrin alpha-3 (ITGA3) (Table 4). To further confirm the higher integrin expression in HPaStec sEVs, Western blot analyses were carried out using representative integrin α and β antibodies. These results again confirmed that both integrins were highly expressed in HPaStec sEVs compared to the HPSC sEVs (supplemental Fig. S9). Next, we identified well-known exosomal markers enriched in HPSC sEVs, including programmed cell death 6-interacting protein (PDCD6IP) or Alix, tumor susceptibility gene, syntenin 1 (SDCBP), Annexin 7 (ANAX7), flotillin 1 and 2 (FLOT1 and FLOT2), annexin 11 (ANAX11), and heat shock 70 kDa protein (HSPA1A/HSPA1B). On the other hand, Ezrin (EZR) and moesin (MSN) were contained at higher levels in HPaStec sEVs (supplemental Table S3).

**Table 2:** Top 10 proteins differentially expressed in HPSC sEVs compared to HPaStec sEVs

Gene symbol(s)	HPSC-HPaStec log_2_ ratio	Standard deviation	Adjusted *p*-value
CSE1L	11.25	6.16	8.55E-03
H2AC14;H2AC12;H2AJ	9.02	4.97	1.20E-02
MACROH2A1	8.2	4.58	4.96E-02
H2AZ2;H2AZ1	8.13	4.48	2.94E-02
H2AC6;H2AW;H2AC4	7.78	4.3	4.41E-02
H1	7.6	4.31	3.85E-02
H2BC14;H2BC15;H2BC9	7.16	3.95	1.15E-02
H4C1	6.68	3.73	2.94E-02
H1	6.44	3.57	2.94E-02
BANF1	5.93	3.31	3.23E-02

**Table 3:** Annexins found in sEVs

Gene symbol(s)	HPSC-HPaSteC Log_2_ ratio	Standard deviation	Adjusted *p*-value
ANXA11	2.45	1.35	4.96E-02^a^
ANXA7	2.08	1.17	2.83E-02^a^
ANXA3	−0.65	0.83	5.55E-01
ANXA2;ANXA2P2	−0.46	0.27	1.11E-01
ANXA10	0.46	0.39	4.53E-01
ANXA5	0.69	0.41	7.43E-02
ANXA1	0.97	0.60	1.24E-01
ANXA6	1.04	0.62	7.71E-02
ANXA4	1.26	0.73	6.80E-02

a indicates differentially expressed between HPSC and HPaStec.

**Table 4:** Integrins found in sEVs

Gene symbol(s)	HPSC-HPaSteC Log_2_ ratio	Standard deviation	Adjusted *p*-value
ITGA2	−3.22	1.77	8.55E-03^a^
ITGA4	−3.52	1.94	1.30E-02^a^
ITGA5	−3.05	1.72	8.20E-02
ITGB5	−2.63	1.57	1.29E-01
ITGB3	−2.58	1.52	1.24E-01
ITGB1	−2.57	1.42	2.47E-02^a^
ITGAV	−2.35	1.33	8.59E-02
ITGB6	−-2.22	1.23	1.96E-02^a^
ITGA3	−2.21	1.23	2.94E-02^a^
ITGA6	−1.25	0.84	1.70E-01
ITGA1	−0.28	0.37	5.55E-01

a indicates differentially expressed between HPSC and HPaStec.

We next performed pathway enrichment analyses of the 87 differentially expressed protein groups using the Enrichr R package (41). Top significantly enriched pathways using Gene Ontology: Biological Process (GO:BP) were consistent with exosome enrichment and included extracellular matrix organization (*P*_adj_ = 3.01 × 10^−04^), multivesicular body assembly (*P*_adj_ = 4.63 × 10^−04^), and cell-matrix adhesion (*P*_adj_ = 3.37 × 10^−03^) (supplemental Fig. S10, *A* and *C* and supplemental Table S3). Top significantly enriched pathways using Gene Ontology: Cellular Component also included extracellular or cell membrane pathways, such as focal adhesion (*P*_adj_ = 2.14 × 10^−14^), filopodium (*P*_adj_ = 9.33 × 10^−04^), and phagocytic vesicle (*P*_adj_ = 2.75 × 10^−03^) (supplemental Fig. S10, *B* and *D* and supplemental Table S3). GO:BP pathway analyses showed endosomal sorting complexes required for transport (ESCRT) complex disassembly and multivesicular body organization and assembly in HPSC sEVs, whereas HPaStec sEVs had elevated cell adhesion and extracellular organization pathways. The ESCRT pathway contained membrane proteins that ubiquitinate and promote the internalization of sEVs within the multivesicular endosome (56). The GO:BP analysis of HPSC sEVs identified an abundance of differentially expressed proteins associated with the lysosomes, a major compartment of protein turnover in the cells. Additionally, Gene Ontology: Cellular Component analysis identified focal adhesion pathway enrichment (*P*_adj_ = 1.31 × 10^−14^), which has a central role in regulating cell signaling (57), as well as a higher abundance of membrane proteins in both of the sEVs (supplemental Fig. S11, *A* and *B*).

**Fig. 5:**
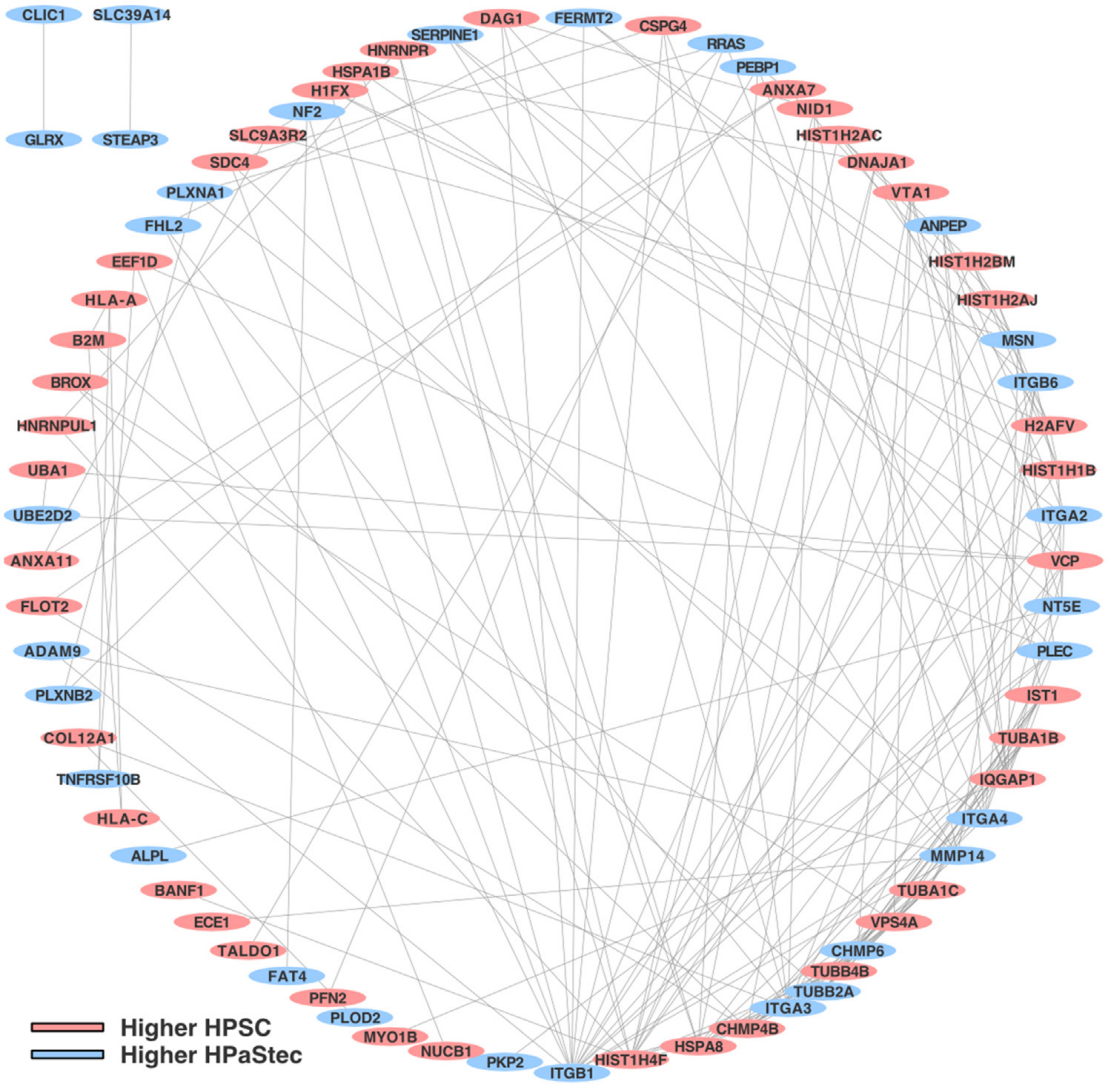
**STRING protein–protein interactions**: Eighty-seven differentially expressed were queried with the STRING protein–protein interaction database. Seventy-three proteins with a minimum interaction score of 0.400 were identified. Notably, 69 were connected in a large network with ITGB1 having the highest degree of interactions (n = 17).

A known shortcoming of pathway enrichment is that it does not consider interactions between pathway members (58).Therefore, we hypothesized an interaction-based approach would capture additional relationships beyond what was found with pathway enrichment. To test this premise, we queried the 87 differentially expressed protein groups with the STRING protein–protein interaction database (44). We used the first protein of the protein group if there was more than one present, and we required at least medium confidence interactions (minimum score 0.400). The resulting network had significantly more interactions than expected (STRING protein-protein interaction enrichment *p*-value < 1.0 E^−16^), and from this, we found 69 protein nodes shared edges in a large sub-network (Fig. 5) with ITGB1 having the highest degree or number of connections (n = 17; supplemental Table S3). These results suggest a commonality between differentially expressed protein groups and may point to a deeper biological process driving the HPSC-HPaStec differences.

### CSE1L is Expressed in PDAC-Associated Pancreatic Stellate Cell sEVs and is Associated with Poor Survival

**Fig.6:**
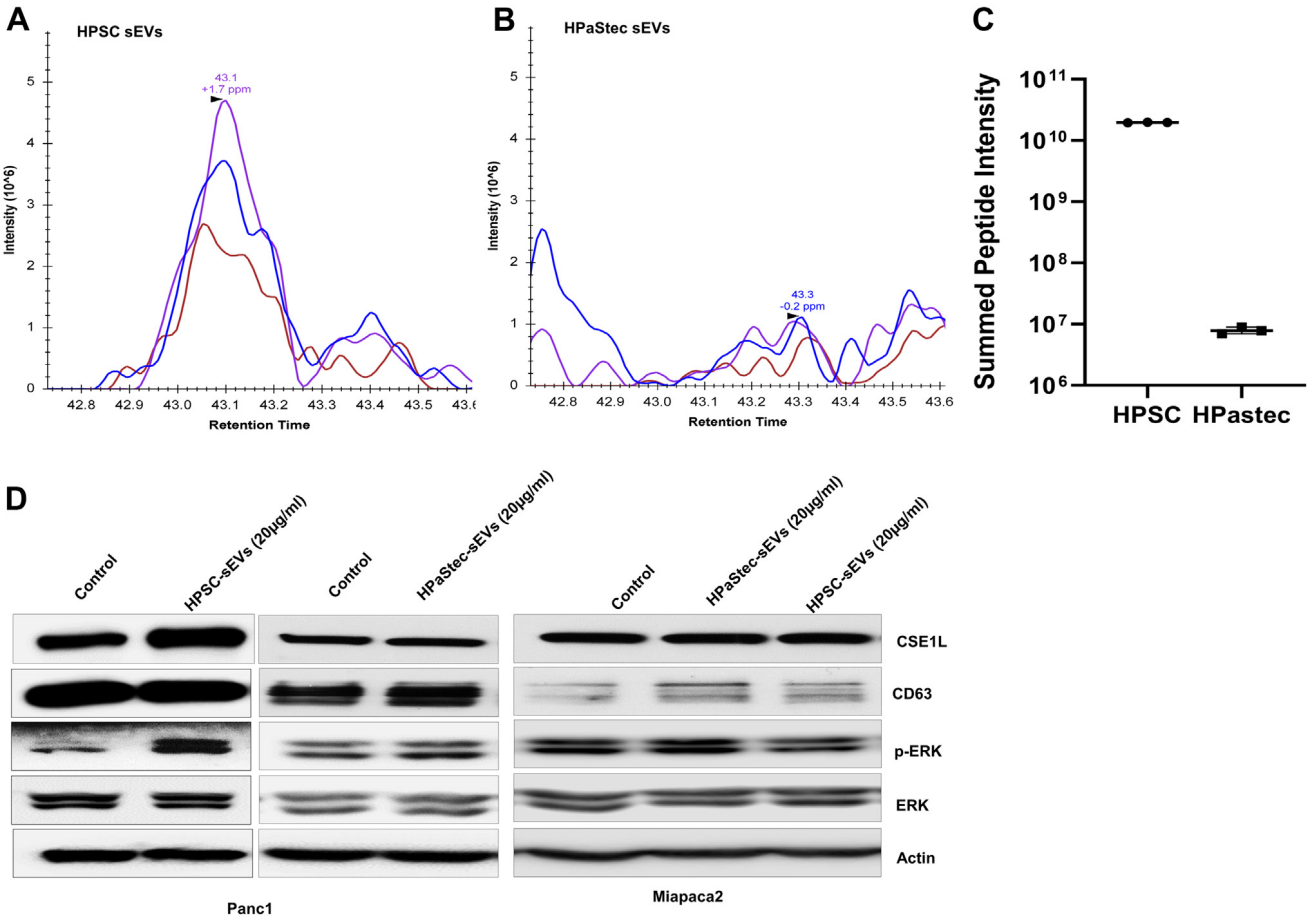
**LC-MS/MS Identification of CSE1/XPO2**: *A* and *B*, relative quantitation with Extracted Ion Chromatograms of the peptide, AADEEAFEDNSEEYIRR, from CSE1L_HUMAN protein shows increased intensity in HPSC sEVs compared to HPastec sEVs. *C*, relative quantitation for CSE1L_human protein. Sum of normalized signal intensity of all identified peptides of CSE1L protein for each sample. *D*, Panc1 or Miapaca2 cells were treated with 20 μg/ml HPSC-sEVs or HPaStec sEVs for 72 h before Western blot analysis using antibodies against CSE1L, CD63, p-ERK, and ERK. Actin was used as a loading control. HPSC, human pancreatic stellate cell; LC-MS/MS, liquid chromatography–tandem mass spectrometry; sEV, small extracellular vesicle.

We have identified specific protein markers that were highly elevated in HPSC sEVs, and among them, CSE1L, a human homolog of CSE1 that is a yeast chromosome segregation protein (59), is preferentially accumulated in HPSC sEVs. CSE1L was dramatically increased in HPSC sEVs (log_2_ ratio = 11.25, *P*_adj_ = 0.009; Tables 2 and S3). CSE1L/XPO2_HUMAN was identified by the peptide, AADEEAFEDNSEEYIRR, with a mass measurement accuracy of 4.36 ppm (see sequence interpretation in supplemental Fig. S12*A, B*). For validation, relative quantitation with extracted ion chromatograms of the isotopes of the intact peptide and the sum of normalized signal intensity of all identified peptides of the CSE1L protein for each sample showed increased intensity in HPSC sEVs compared to HPaStec sEVs (Fig. 6, *A*–*C*, Supplemental Fig. S12). CSE1L expression was also significantly higher in the HPSC sEV fractions than the HPaStec sEVs (Fig. 2*A*) *via* Western blot analyses. CSE1L expression is correlated with poor overall survival in other cancer types (56), but to our knowledge, it has not been investigated for PDAC. Given the dramatic increase of CSE1L in HPSCs, we hypothesized our findings may be relevant to outcomes of patients with PDAC. Therefore, we identified 174 patients with PDAC from the Cancer Genome Atlas (TCGA) (57) with CSE1L gene expression measurements and clinical outcome data, and we stratified these patients by median CSE1L expression. Kaplan-Meier survival analysis revealed that higher expression of CSE1L was associated with a significant decrease in survival (log-rank *p* value = 9.95 E^−4^; supplemental Fig. S13).

### CSE1L Expression and ERK Signaling in Panc1 Cells Upon HPSC sEVs Treatments

Next, to investigate the effect of HPSC sEVs and HPaStec sEVs on CSE1L expression, HPSC sEVs (20 μg/ml) were cocultured with Panc1 or Miapaca2 cells for 72 h prior to Western blot analyses using CSE1L, CD63, and β-actin antibodies. In addition, to determine if sEVs treatment to the cells induce the cell survival signaling, we have assessed the phosphorylated ERK signaling after sEVs treatment. The Western blots revealed elevated CSE1L expression and increased ERK signaling (Fig. 6*D*) in Panc1 cells but not in Miapaca2 cells upon treatment with HPSC sEVs. On the contrary, primary HPaStec sEVs did not modulate the expression of CSE1L or ERK signaling (Fig. 6*D*) in any of the cancer cells.

## Discussion

The PDAC tumor microenvironment is largely composed of stromal cells, including stellate cells. Although activated stellate cells, and sEVs secreted from them, are known to contribute to the aggressive biology of PDAC (2, 3, 24, 60, 61, 62), much remains to be learned regarding stromal-derived sEVs in pancreatic cancer. Here, we have performed a unique qualitative and quantitative comparison of the proteome found in the sEVs derived from stellate cells isolated and cultured from normal pancreas (HPaStec) and PDAC tumors (HPSC). Key findings from this study include the following: (1) overall trend in total EV concentration that increases in HPSC cells and a significantly higher mean sEVs sizes in HPSCs; (2) efficient uptake, that is dependent upon sEV surface proteins, of both HPaStec- and HPSC-derived sEVs into both normal and pancreatic cancer epithelial cells; and (3) the absence of significant *in vitro* effect of either normal and tumor-derived sEVs on pancreatic cancer cell survival or growth; and (4) detailed comparative proteomics analysis comparing normal and tumor stellate cell–derived sEVs identified 87 differentially expressed proteins suggesting that each is biologically distinct from each other. Together, these findings support sEVs as warranting further study as potential tools for drug delivery across different biological barriers and use as next generation therapeutics.

In our study, we isolated and characterized sEVs from HPSC and HPaStec cells in terms of morphology, size distribution, and concentration. The biology of both cell types are different and that may contribute towards differences in sEVs generations. Previous coculture experiments have identified that soluble factors from HPSC cells stimulate signaling pathways involved in proliferation and survival of pancreatic cancer cells *in vitro* and *in vivo* (29).

Investigators have demonstrated crosstalk *via* sEVs between stromal and cancer cells with demonstration of miRNAs for chemokine ligands proposed as a potential driver of proliferation and survival of pancreatic cancer cells (60). Our results suggest that HPSC-secreted sEVs, in distinction from the soluble factors in the conditioned medium, do not influence the *in vitro* proliferation, apoptosis, or motility of both tumor and stellate cells. Efficient uptake of sEVs was demonstrated in culture conditions suggesting that observed differences *versus* previous reports (29) could be related to the unique stellate cells used in the assays or differences in the sEV isolation techniques.

Nevertheless, stellate cell sEVs contain a robust proteome that harbors surface and cargo proteins critical to their biology and our investigation of proteome offers unique opportunities to exploit sEVs as biomarkers or drug delivery vehicles. Small extracellular vesicular proteins can be more specific than secretory proteins in cells (23, 63) in terms of identifying diagnostic biomarkers related to cancers. As previously reported by Melo SA *et al.* (24), GPC1 was specifically enriched on cancer cell–derived exosomes and showed high specificity over CA-199 or serum-free GPC1 (100% vs 79.49% vs 82.14%) that distinguish noncancer patients from pancreatic cancer patients. With respect to drug delivery, we demonstrated that membrane proteins on HPSC sEVs may be essential for sufficient exosomal entry to normal and cancer cells, as documented by recent studies (21, 61). In other words, the role of membrane proteins in uptake of small extracellular vesicles may be relevant to ongoing studies using sEVs as vehicles to improve drug delivery efficacy and reduce toxicity of chemotherapeutic drugs (20).

To the best of our knowledge, this report is the first to compare biological and proteomic analysis between cancer-associated HPSC sEVs and normal pancreatic stellate cell sEVs. Consistent with exosomal enrichment, the protein groups we identified shared a large degree of overlap with ExoCarta (42), a database of exosomal proteins, RNAs, and lipids. We identified 87 differentially expressed protein groups, including APNEP, MMP14, and CSE1L, between HPSC and HPaStec sEVs. Our results (Tables 2 and S3) showed differential expression of several histone protein groups. Strikingly, many DNA-binding proteins, such as H2A, H2B, H3, and H4, were enriched in HPSC sEVs; although histones are not commonly regarded as proteins associated with extracellular vesicles, histones were seen to be enriched in sEVs in a murine B16 melanoma model and in primary HPSC exosomes (64, 65). Several studies have also shown that sEVs contain chromosomal DNA fragments, indicating that exosome secretion maintains cellular homeostasis by removing harmful cytoplasmic DNA from cells (66, 67, 68, 69, 70). In the current study, elevated DNA-binding proteins were observed in HPSC sEVs which may be indicative of cell growth and protein production. More detailed studies with pancreatic stellate cell sEVs isolated from PDAC tumors are needed to better understand the source of enriched histone proteins in sEVs and their pathobiological roles.

Among annexin group of proteins, ANAX 7 and ANAX 11 are differentially expressed in HPSC sEVs when compared to those from HPaStec. Annexins have several cellular functions, including cell migration, proliferation, and apoptosis (71), and changes in the expression of individual annexins have been observed in cancers (72), thus implicating that differential expression of annexins in HPSC sEVs may account for PDAC development. HPSC sEVs are also enriched with syntenin (SDCBP), and it was shown that microvesicle formation is regulated through the syndecan heparan sulfate proteoglycans and their cytoplasmic adaptor, syntenin (73). Pathway enrichment of the differentially expressed protein groups was again consistent with exosome enrichment and included extracellular matrix and cellular membrane pathways. Integrin β1 (ITGB1) found in sEVs had highest protein–protein interactions, suggesting it may serve as a hub of exosome cargo delivery and organ-specific intracellular signaling (21, 65).

CSE1L, a microvesicle membrane protein also known as Exportin, is located on the 20q13 locus. Although primarily expressed in the nucleus, CSE1L is also found in sEVs (74). CSE1L is involved in forming microtubule assembly and apoptosis, and it functions as a nuclear transporter and transcriptional regulator (75, 76). CSE1L regulates Ras-induced ERK phosphorylation, microvesicle generation, and tumor metastasis (59). It is amplified or highly expressed in pancreatic cancers (77) and a variety of other cancers (74). Knockdown of CSE1L has been shown to inhibit tumorigenesis and induce apoptosis (78). Here, we report that CSE1L was significantly elevated in HPSC sEVs and may contribute towards the cancer signaling pathway in PDAC. Moreover, coculture of HPSC sEVs with Panc1 cells increased ERK phosphorylation and CSE1L expression. CSE1L overexpression triggered microvesicle generation and tumor progression, thereby regulating the Ras-ERK signaling pathway (59). Our secondary analysis of the Cancer Genome Atlas data revealed that higher gene expression of *CSE1L* is significantly associated with reduced survival in PDAC (log-rank *p* value = 9.95 E^−4^). This result is consistent with findings with other cancer types and hints at a translational avenue for our results. CSE1L-triggered microvesicle generation might explain the higher exosome content in CSE1L-enriched HPSC sEVs than HPaStec sEVs (59).

The limitations of our present study include reliance on sEV isolation exclusively from cultured cell lines, not from direct patient samples. Therefore, further studies are needed to understand the source, association, and secretion of elevated levels of CSE1L from stellate cell sEVs adjacent to a PDAC tumor and the relevant clinical importance in cancer therapy and diagnosis. Future work is planned to further investigate the relationship between exosomal CSE1L protein expression and PDAC patient outcomes. In future, it would be also interesting to determine whether stellate cells–associated sEVs can be used as prognostic markers of PDAC to validate the current findings.

In summary, we have successfully identified differentially expressed proteins in HPSC sEVs isolated from PDAC-associated stellate cells and in sEVs isolated from normal pancreases. Our results suggested that HPSC sEVs are biologically different from HPaStec sEVs and may provide an advantageous microenvironment to the pancreatic cancer cells and may potentially be targeted as a cargo vehicle for the safe delivery of drugs or other biological materials to the cancer cells. Additional studies are required to elucidate the role of cancer-associated stellate cell sEVs in PDAC progression, which would lead the way to therapeutic intervention targeting tumor stroma.

## Data Availability

The mass spectrometry proteomics data have been deposited to the ProteomeXchange Consortium *via* the PRIDE (79) partner repository with the dataset identifier PXD030077 and 10.6019/PXD030077.

This article contains supplementary figures and Tables.

## Supplemental data

This article contains supplemental data.

## Conflict of Interest

Dr John M. Koomen is partially funded by BMS for a different project unrelated to this work. Dr Jason B. Fleming is a scientific advisor/consultant in the following companies: Glycosbio Food Sciences, Inc, Biopath Holdings Inc, Natera, Panther Therapeutics Inc, and an independent contractor in MyCareGorithm. Other authors have no conflict of interests with the contents of the article.
