# Supplementary material for: A comparative Proteomics Analysis Identified Differentially Expressed Proteins in Pancreatic Cancer–Associated Stellate Cell Small Extracellular Vesicles
Source: Mol Cell Proteomics. 2022 Nov 2;21(12):100438. doi: 10.1016/j.mcpro.2022.100438 (PMC9792568; doi:10.1016/j.mcpro.2022.100438)
Supplement: Supplementary figures [file mmc2.pdf]

Supplementary Data

Title: **A comparative proteomics analysis identified differentially expressed proteins in pancreatic cancer–associated stellate cell small extracellular vesicles**

Bhaswati Sarcar[1], Bin Fang[2], Victoria Izumi[2], Yury O. Nunez Lopez[3], Alexandra Tassielli[1], Richard Pratley[3], Daniel Jeong[4], Jennifer B. Permuth[1,5], John M. Koomen[2], Jason B. Fleming[1*], Paul A. Stewart[6*]

[1]Department of Gastrointestinal Oncology, [2]Proteomics and Metabolomics Core Facility, H. Lee Moffitt Cancer Center and Research Institute, Tampa, FL, [3]Translational Research Institute, Advent Health, Orlando, FL, [4]Department of Diagnostic and Interventional Radiology, [5]Department of Cancer Epidemiology, [6]Department of Biostatistics and Bioinformatics, H. Lee Moffitt Cancer Center and Research Institute, Tampa, FL

Supplementary Data include:

- Supplementary Figures S1-S13
- Supplementary Video S1
- Supplementary tables S1, S2 and S3 (separate format)

Supplementary Figures

Fig. S1: **Growth rate and morphology of HPSC and HPaStec cells:** 5X10^6 of either HPSC or HPaStec cells were seeded in T150 cm^2 flasks to assess their growth rate by Trypan blue (Gibco) exclusion method at 24 and 48hrs as shown in the graph (a) (Average of three independent experiments). Representative cell numbers at 24 and 48hrs. were shown in the table. There is no significant difference (HPSC vs. HPaStec cells, unpaired t test, p=0.80 and 0.77 at 24 and 48hrs.) in growth rate of HPSC and HPaStec cells as determined by the cell number and confluency. (b, c) Cellular morphology of both the cells were captured using Incucyte S3 Live Imager with 10x magnification in-phase channel at 48hrs (scale bar=50μm).

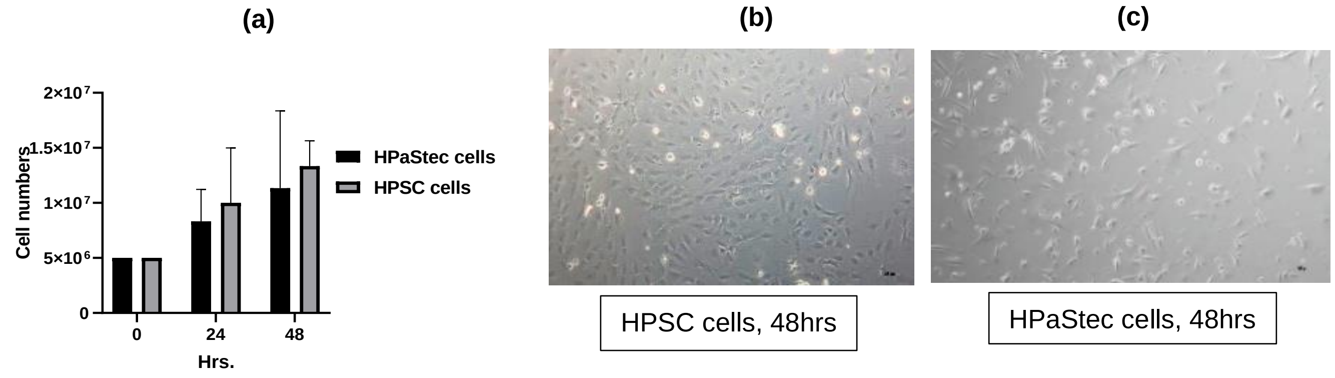

Cell type	Seeding density	Mean cell no. (24 hrs.)	Mean cell no. (48 hrs.)
HPaStec	5X10^6	8.33X10^6	1.13X10^7
HPSC	5X10^6	1X10^7	1.33X10^7

Fig. S2: **sEVs imaging by image cytometry**: Imaging of APC-conjugated CD63 (Red) of HPSC and HPaStec sEVs by Image Stream software as described in the methods section.

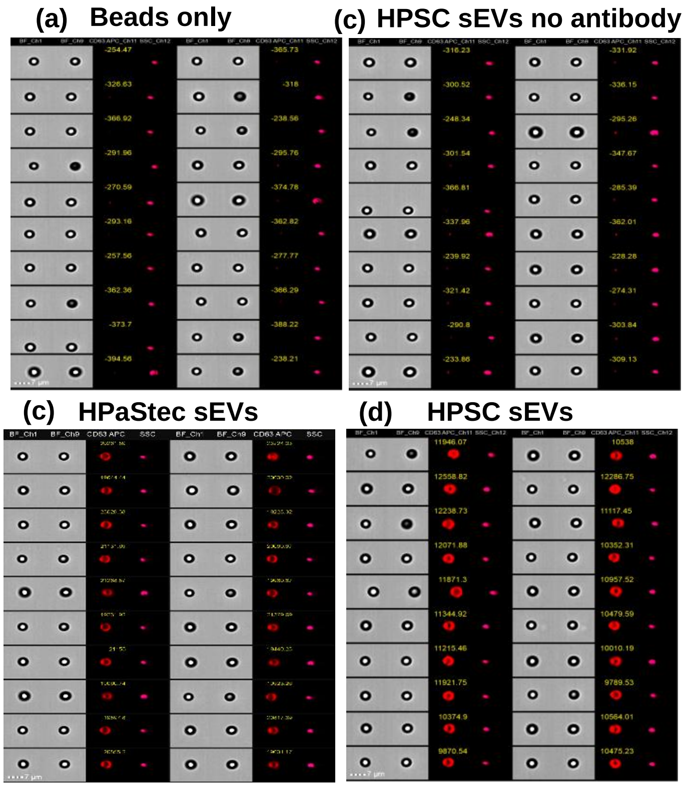

Fig. S3: Cell proliferation assay. (a, b, c) HPNE, Panc1 and Miapaca2 cells were seeded separately in 96 well plates (1,000 cells/well). Next day, the cells were treated with vehicle control (PBS) or either with HPSC or HPaStec sEVs in a dose dependent manner as indicated for 24hrs. Also, (d) Panc1 (1,000 cells/well) or (e) Miapaca2 cells (1,000 cells/well) were seeded in 96 well plates (day 0) overnight. On the next day, cells were treated with vehicle control (PBS) or 40µg/ml HPSC or HPaStec sEVs**,** for 72hrs. (f) Panc1 (1,000 cells/well) or (g) Miapaca2 cells (1,000 cells/well) and stellate cells (1,000 cells/well) were mixed and seeded at 1:1 ratio in 96 well plates (day 0) overnight. On the next day, cells were treated with vehicle control (PBS) or indicated amount of sEVs**,** for 24hrs. Analysis was done incubating the cells with CellTiter-Glo Luminescent reagent (Promega). Data were recorded using Flax station 3 plate reader (Molecular Devices), cell viability was normalized to vehicle-treated wells and fit to a sigmoidal dose-response curve using GraphPad Prism 6. All experiments were performed in triplicate and repeated multiple times and the representative data were shown in the figs. The data were statistically analyzed using unpaired t-test and two tailed. *p* values (=non-significant, ns) were indicated in the respective figures. (h) The representative live-cell images of Panc1 and primary HPaStec cells incubated with or without HPSC sEVs as indicated were taken in an Incucyte S3 Live Imager (Sartorius Corporation, NY) with 10X magnification (scale bar=200µm) in-phase channel at 0 and 24hrs.

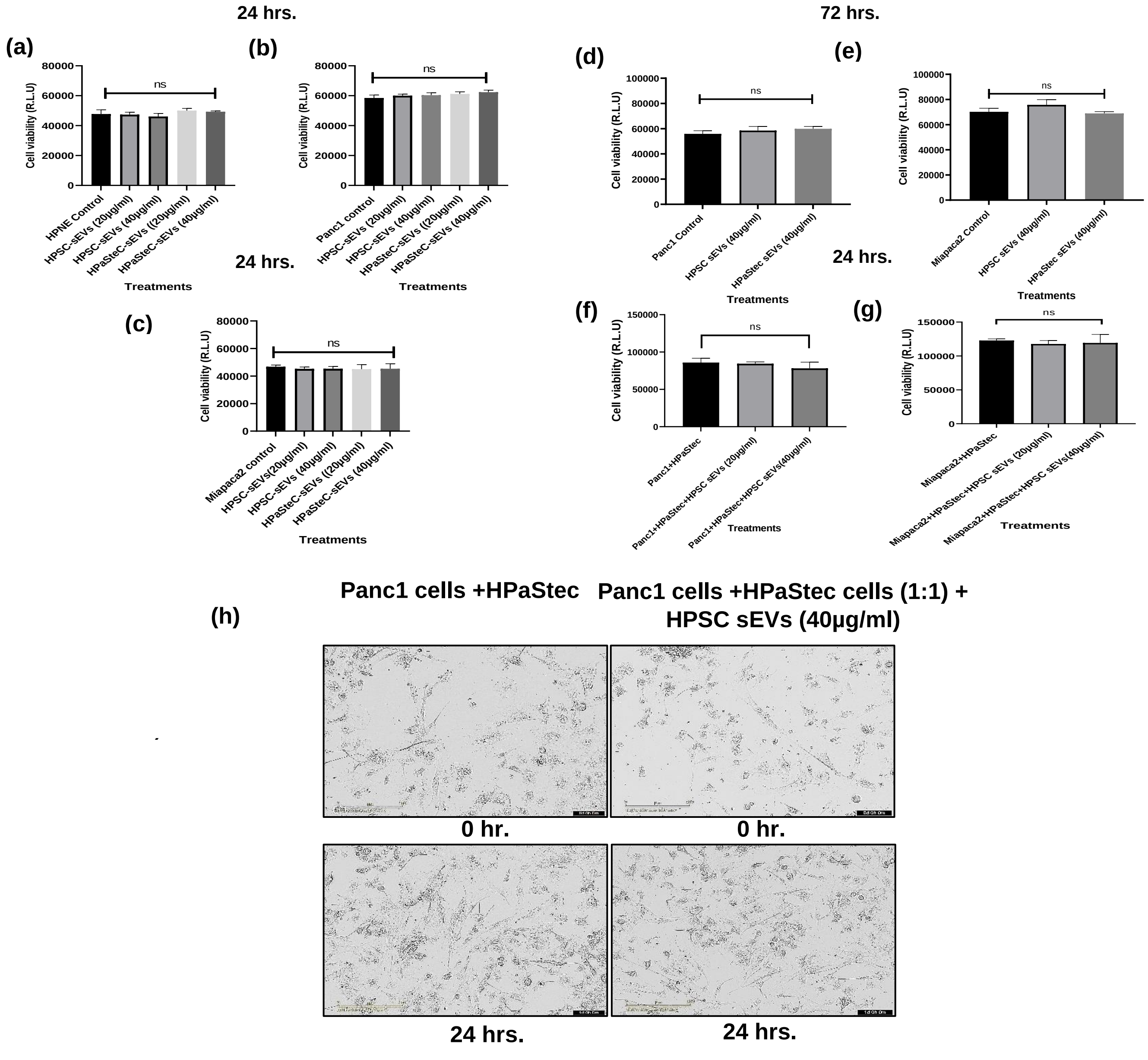

Fig. S4: Apoptosis assays: Either Panc1 or Miapaca2 cells were incubated with HPSC sEVs at 40µg/ml and 80µg/ml for 72hrs. respectively followed by Western blotting to probe for c-caspase 3 and cleaved -PARP (a, b). Next, for caspase 3 and 7 assay, HPSC sEVs were incubated with Panc1 at 40µg/ml for 24hrs followed by immunofluorescence to detect the (c) caspase 3/7 in the cancer cells using Image-iT^{TM} Live Red caspase 3 and 7 detection kit (Invitrogen) following manufacturer's instructions. 1µg sotrastaurin (Selleckchem) was used as positive control to treat the cells. Sytox green stained the live cells, the FLICA (red) represents the caspase 3/7 activated cells and Hoechst (blue) stained the nucleus. (d) HPSC sEVs were incubated with Panc1 at 40µg/ml and 80µg/ml for 24hrs. followed by immunofluorescence to probe for c-PARP (red). β-tubulin antibody (green) was used to stain the cytoskeleton of Panc1 cells and DAPI stained the nucleus. As indicate in both (c) and (d), Images were captured at 63X magnification (scale bar: 25µm) using a Leica SP8 AOBS laser scanning confocal microscope through a 63×/1.4NA Plan Apochromat Oil Immersion Objective Lens (Leica Microsystems CMS GmbH, Germany). (e) Panc1 cells were seeded with HPaStec cells in 1:1 ratio and cultured in respective medium to confluent monolayers as described in the methods section. Next day, a linear wound was generated in the monolayer with a commercially available wound maker (Essen Bioscience, MI). HPSC sEVs (40µg/ml) were added to the cells and time lapse imaging was performed by scratch wound assay analysis module in an Incucyte S3 Live imager (Sartorius Corporation, NY) with 4X magnification in-phase channel. Random images were chosen for measurement at 0,6,12,18 and 24hrs. Experiments were done in quadruplicates and wound confluency (f) and width (g) were plotted in GraphPad Prism.

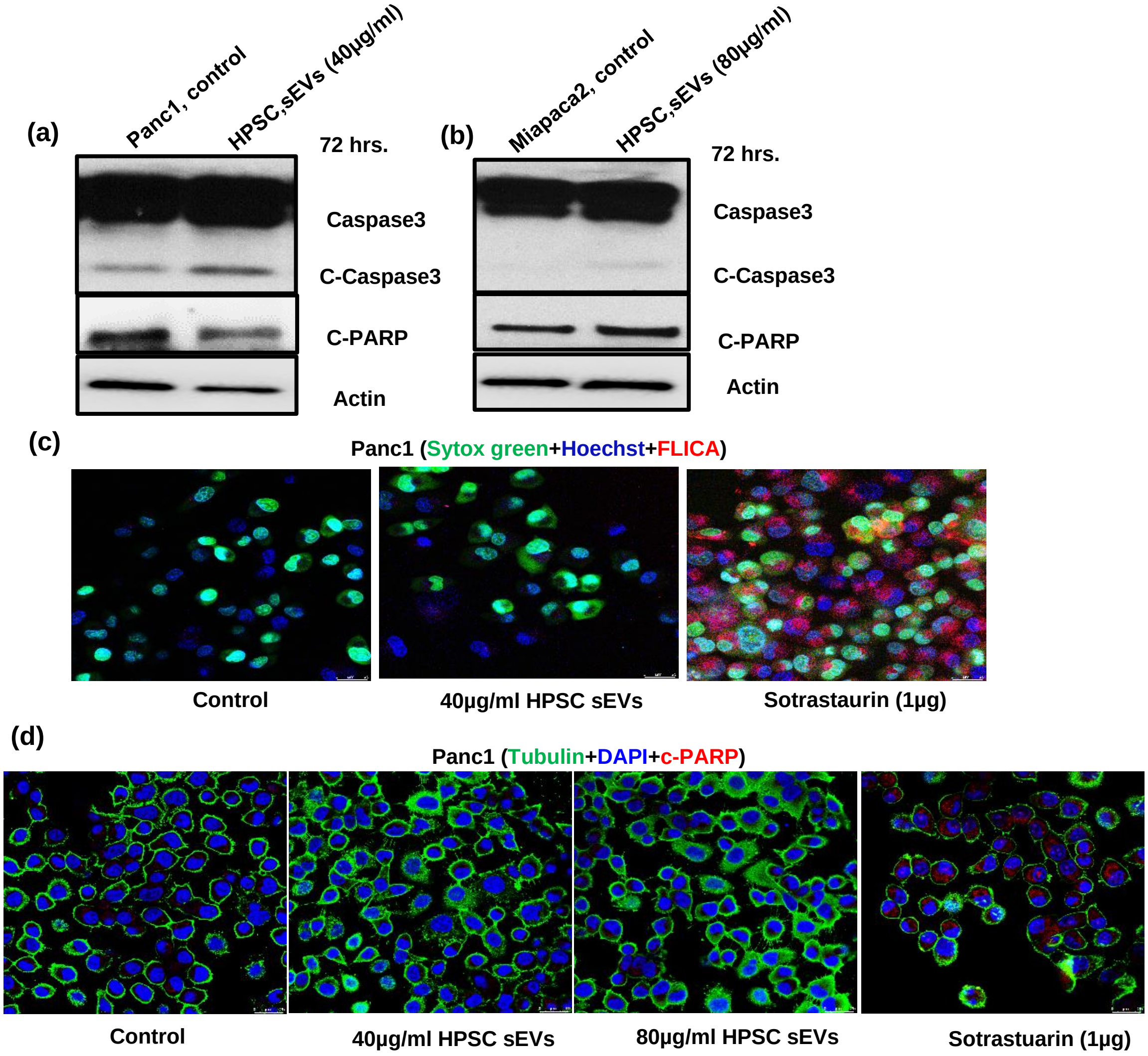

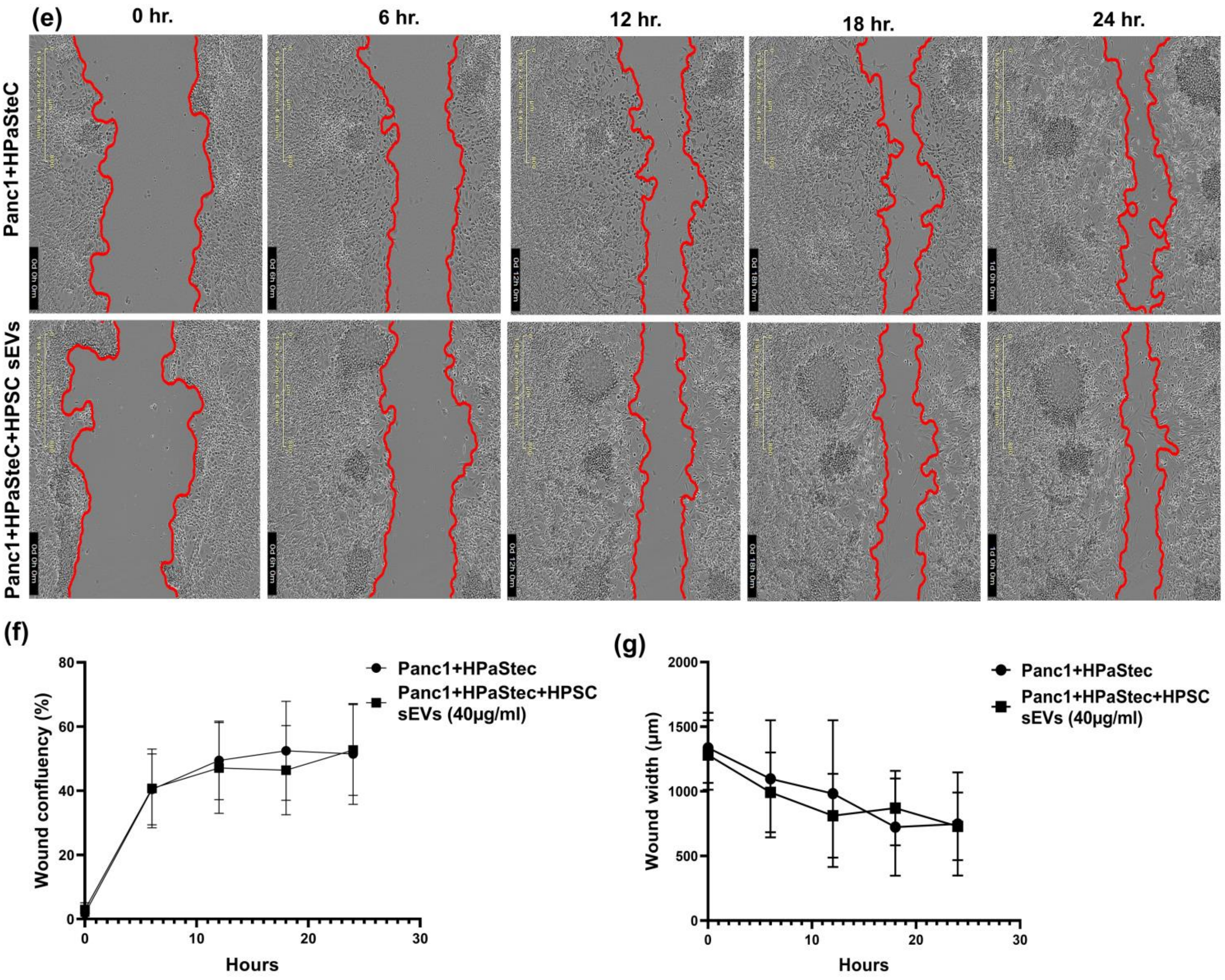
(e)
0 hr.
6 hr.
12 hr.
18 hr.
24 hr.
Panc1+HPaSteC
Panc1+HPaSteC+HPSC sEVs
(f)
Wound confluency (%)
Hours
Panc1+HPaStec
Panc1+HPaStec+HPSC sEVs (40μg/ml)
(g)
Wound width (μm)
Hours
Panc1+HPaStec
Panc1+HPaStec+HPSC sEVs (40μg/ml)

Fig. S5: **Proliferation and cytotoxicity assays**. Five million cells (HPSC and HPaStec) were seeded with respective media without serum and after 48hrs. conditioned media were collected, centrifuged to remove the cellular debris and concentrated and protein contents were measured as described in the methods section. (a, b) Either Panc1 or Miapaca2 cell lines were seeded in 96-well plates at a density of ~4000 cells/well were treated either with 1µg/ml HPaStec or HPSC CM for 48hrs. to determine the proliferation of Panc1 (HPSC CM, ****p<0.0001) and Miapaca2 cells in presence of HPaStec and HPSC CM. Next, for cytotoxicity assay, the cells were treated with gemcitabine (concentration ranging from 0.01–100µM) alone or in combination with 1µg/ml HPaStec or HPSC CM. (c, d) To determine dose-dependent gemcitabine-induced cytotoxicity alone or in combination with HPSC/HPaStec sEVs CM, cell viability and the corresponding EC50 values were determined using the CellTiter-Glo chemiluminescent assay (Promega). Results are the average of three independent experiments.

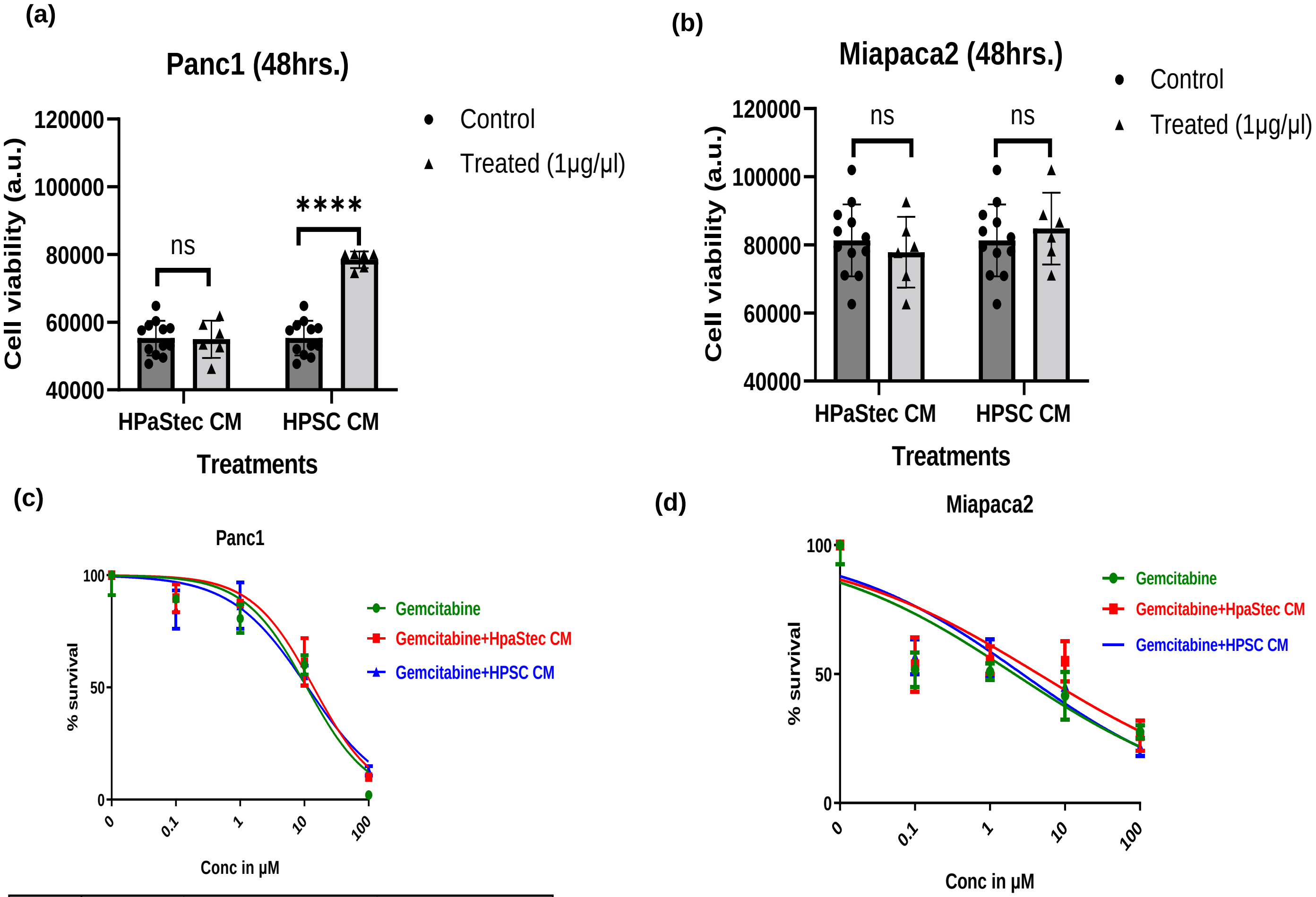

	Gemcitabine	Gemcitabine+HpaStec CM	Gemcitabine+HPSC CM
EC50	10.89	13.91	11.23

	Gemcitabine	Gemcitabine+HpaStec CM	Gemcitabine+HPSC CM
EC50	2.125	4.459	2.703

Fig. S6: Comparison of proteomics data with public database: We intersected our protein identifications with those from Servage et al. In total, 76% of our protein identifications were found in this dataset.

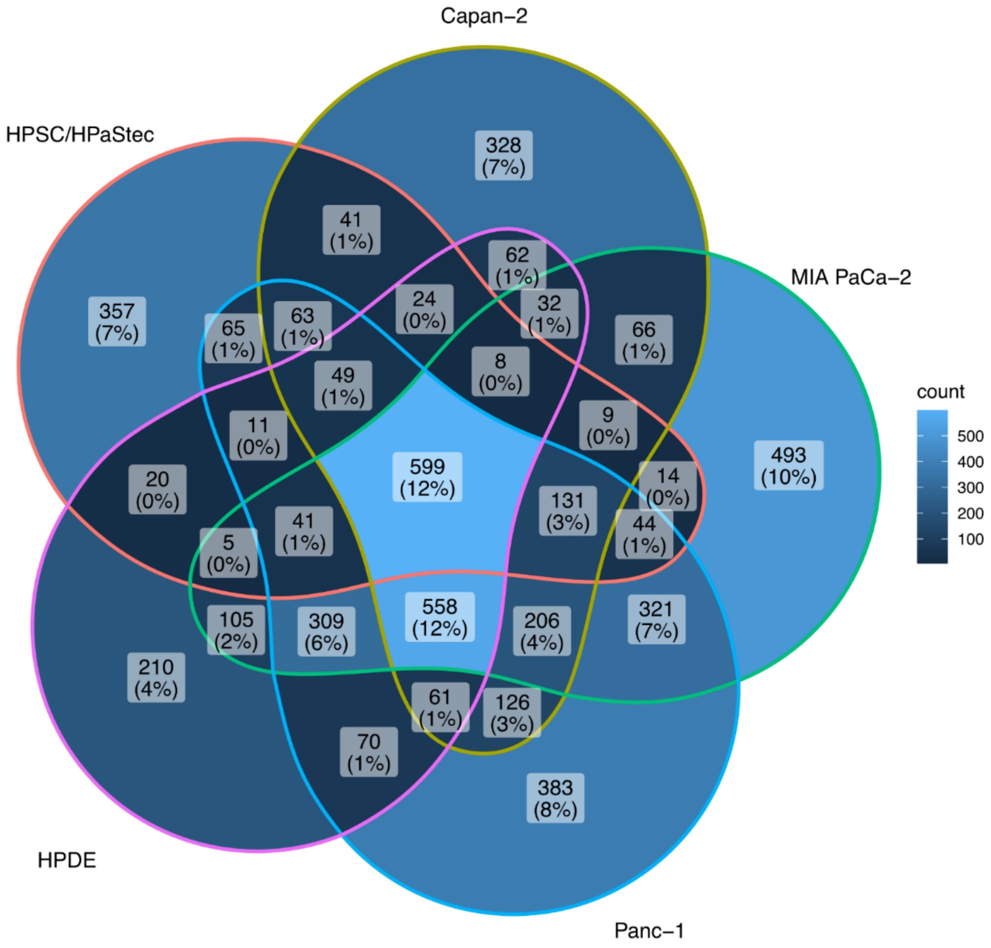

Fig. S7: Quality control for exosome proteomics data was evaluated using boxplots of log2 intensities before (a) and after (b) iterative rank order normalization (IRON). (c) Principal component analysis of exosome protein expression shows samples are separated in the first principal component (PC1) by their cell type of origin.

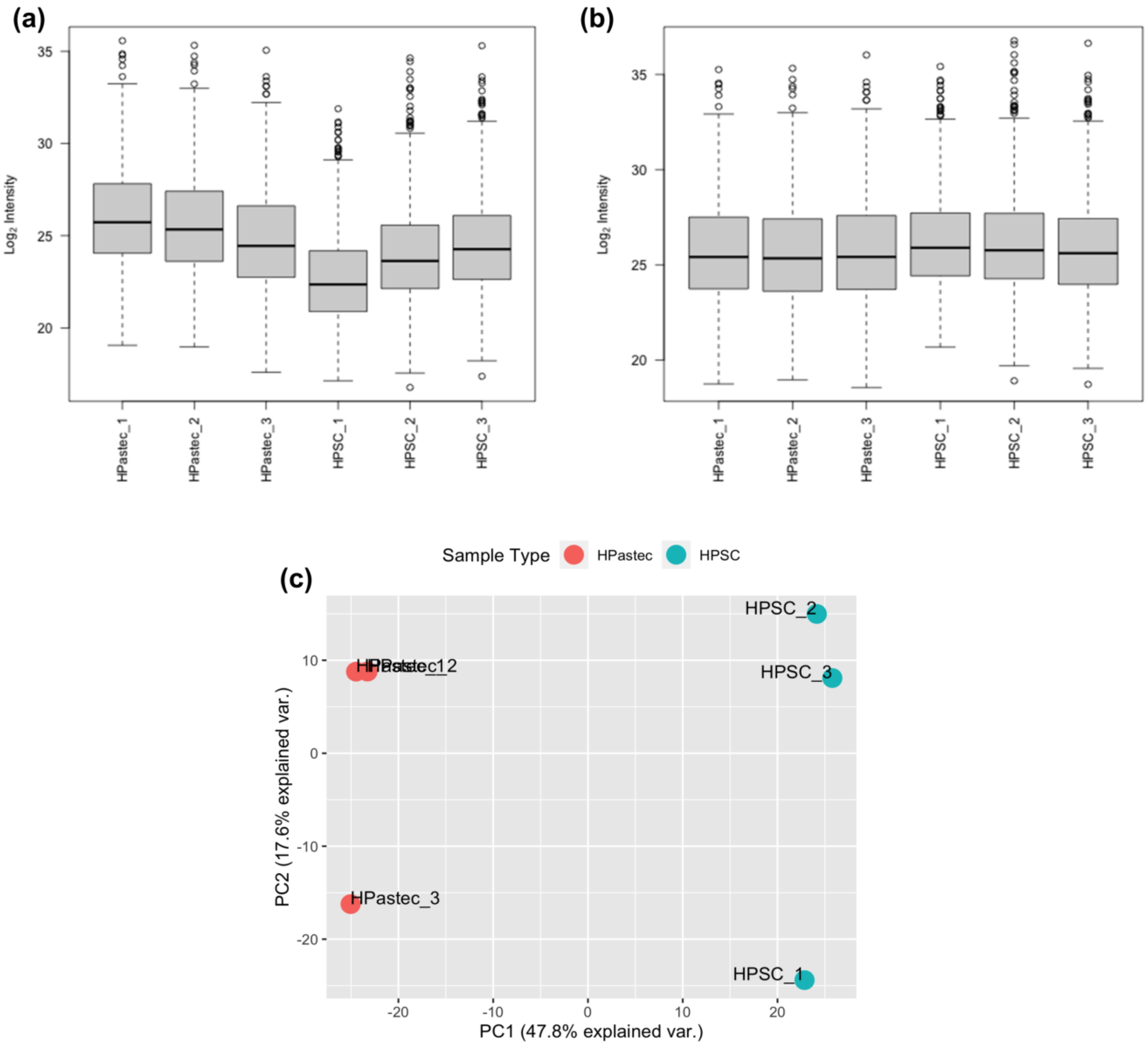

Fig. S8: Protein identification in FBS with Uniprot: We independently processed and ran FBS through LC-MS/MS, searched with the human Uniprot database, and intersected with our results. Protein overlaps between FBS and our findings was minimal (2% of identifications or 30 proteins).

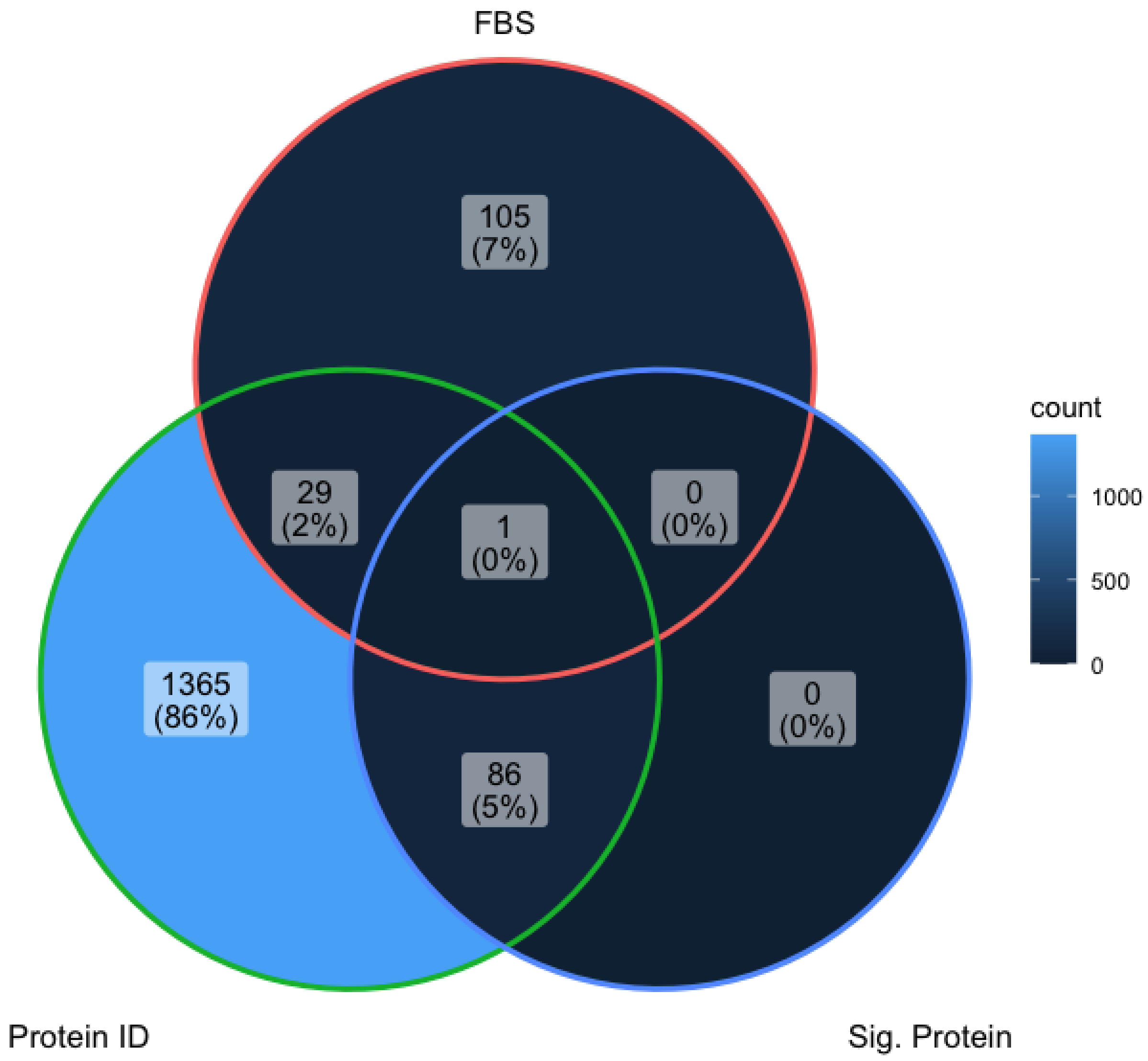

Fig. S9. Integrin α4 expressions. Western blot analysis was performed in HPSC and HPastec sEVs with the parental cells lysates to examine the integrin expression (integrin α4 and integrin β1). 20 μg of total protein was loaded in each lane. Actin is used as loading control for the cell lysates.

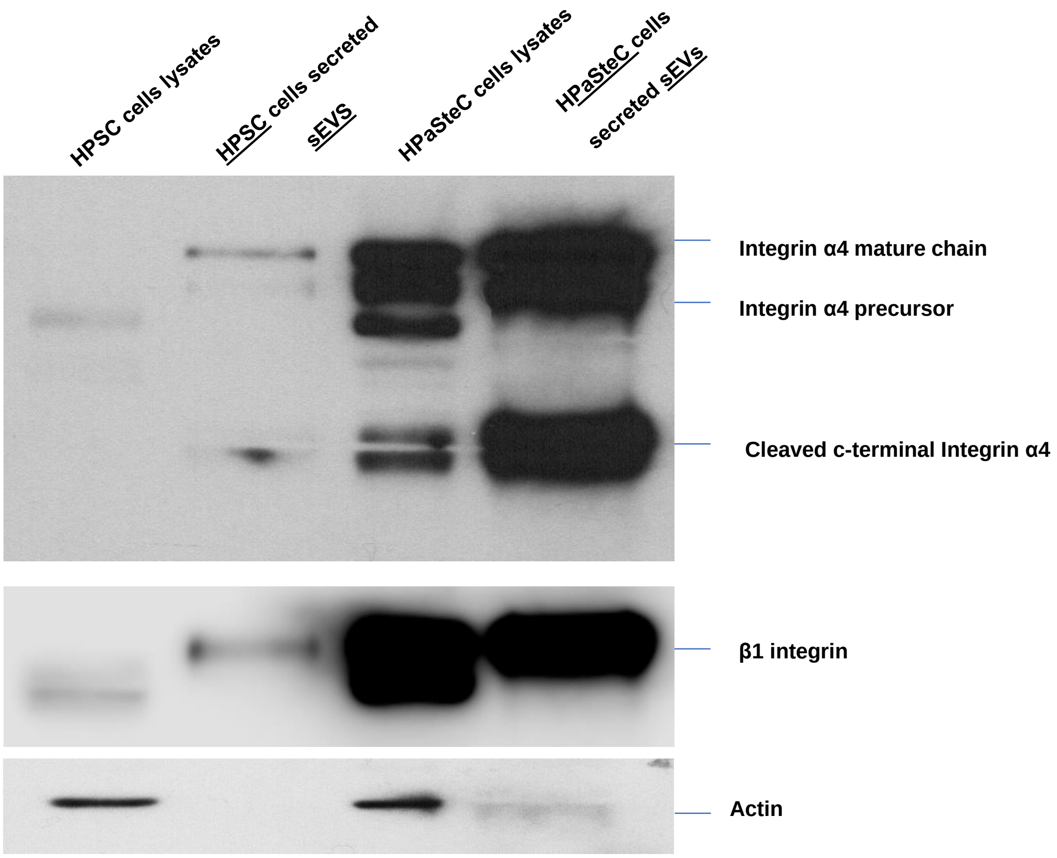

Fig. S10: **Pathway analysis of differential expressed proteins in the sEVs cargo**. (a) The Enrichr R package was used to perform pathway enrichment analysis of the 37 protein groups significantly decreased in HPSC with the GO:BP database. (b) The Enrichr R package was used to perform pathway enrichment analysis of the 37 protein groups significantly elevated in HPSC with the GO: CP database. (c) The Enrichr R package was used to perform pathway enrichment analysis of the 50 protein groups most significantly elevated in HPaStec with the GO:BP database. (d) The Enrichr R package was used to perform pathway enrichment analysis of the 50 protein groups most significantly elevated in HPaStec with the GO:CP database. The dashed red line to the bar plots indicate $p_{adj} = 0.05$.

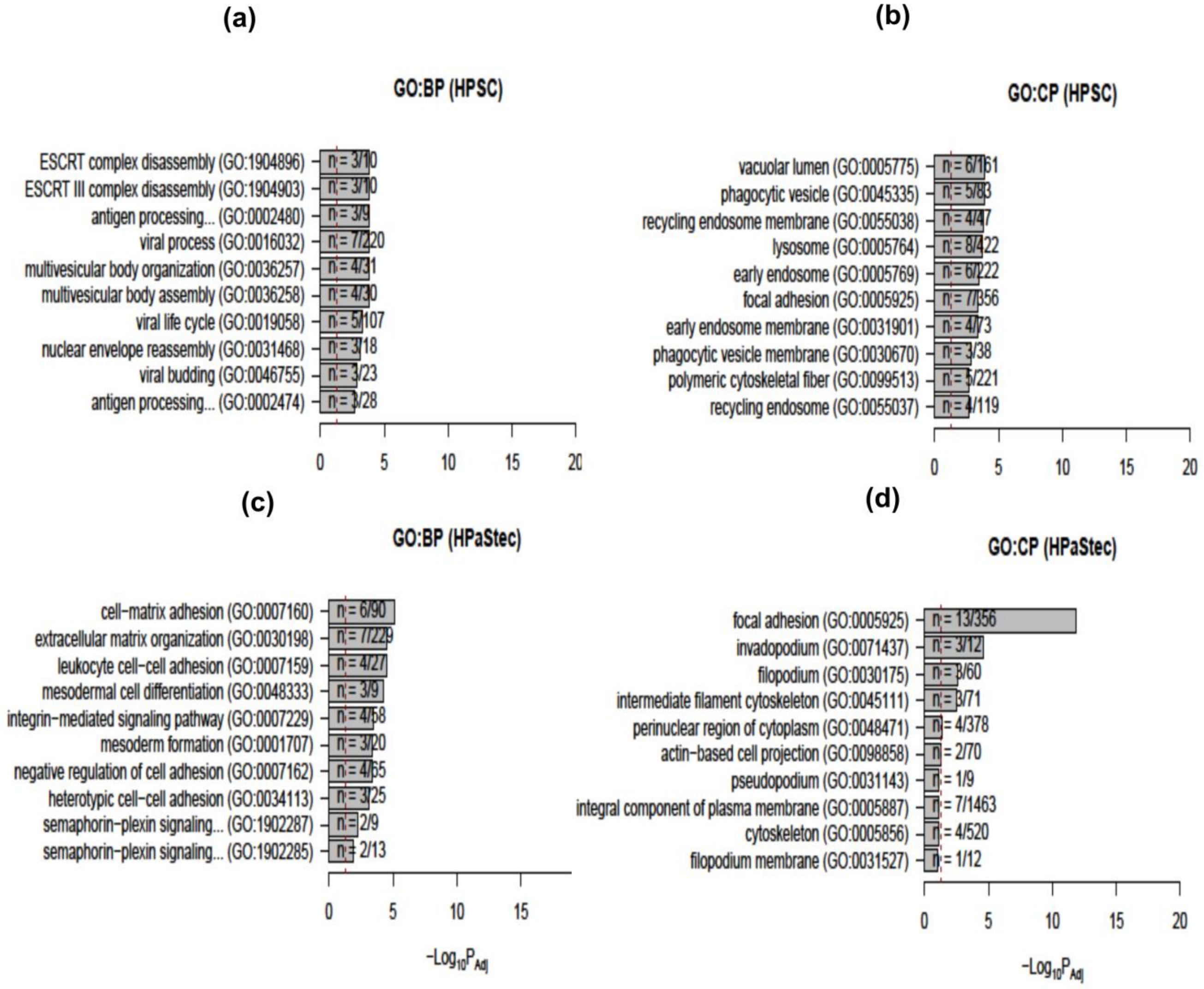

Fig. S11: Pathway analysis of differential expressed proteins in the sEVs cargo. (a) The Enrichr R package was used to perform pathway enrichment analysis of the 87 differentially expressed protein groups with the GO:BP database. Extracellular processes were among the top hits and were consistent with exosome enrichment. (b) The Enrichr R package was used to perform pathway enrichment analysis of the 87 differentially expressed protein groups with the GO:CP database. Extracellular or cell membrane pathways were among the top hits and were consistent with exosome enrichment. The dashed red line to the bar plots indicate p_{adj} = 0.05.

(a)

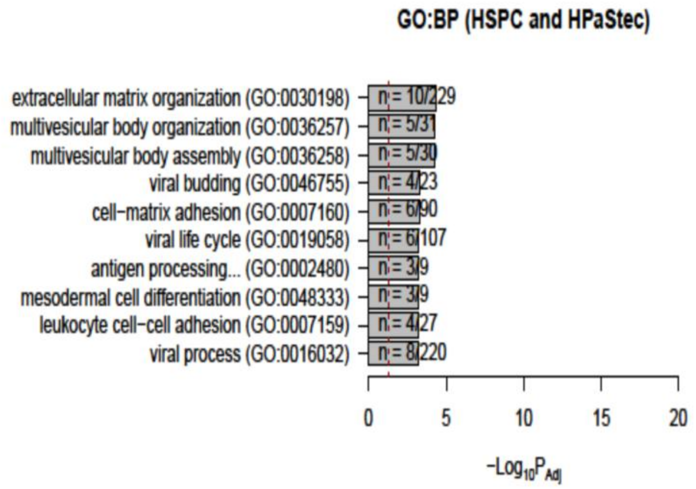

(b)

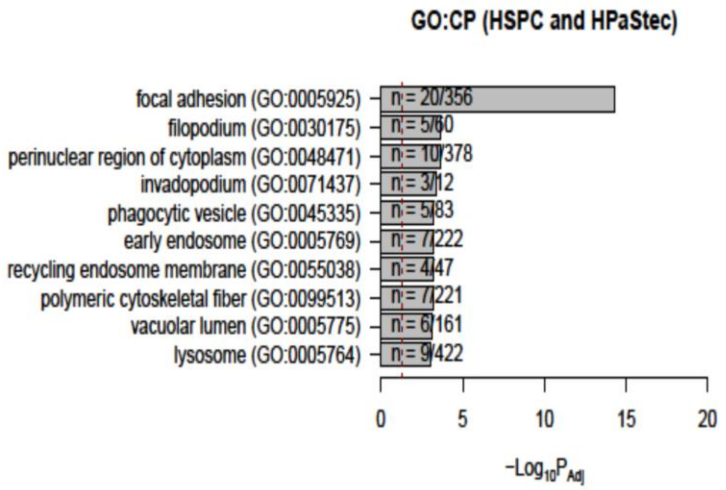

Fig. S12. **LC-MS/MS Identification of CSE1/XPO2**: The LC-MS/MS spectrum represents the identification of AADEEAFEDNSEEYIRR peptide (Map has shown in the figure) from CSE1L/XPO2_HUMAN. The m/z of the precursor was detected at 681.9708 (3+), which represented a mass measurement accuracy of 4.36 ppm. The identification was made with Sequest software (in Proteome Discoverer, Thermo) with an XCorr score of 3.29.

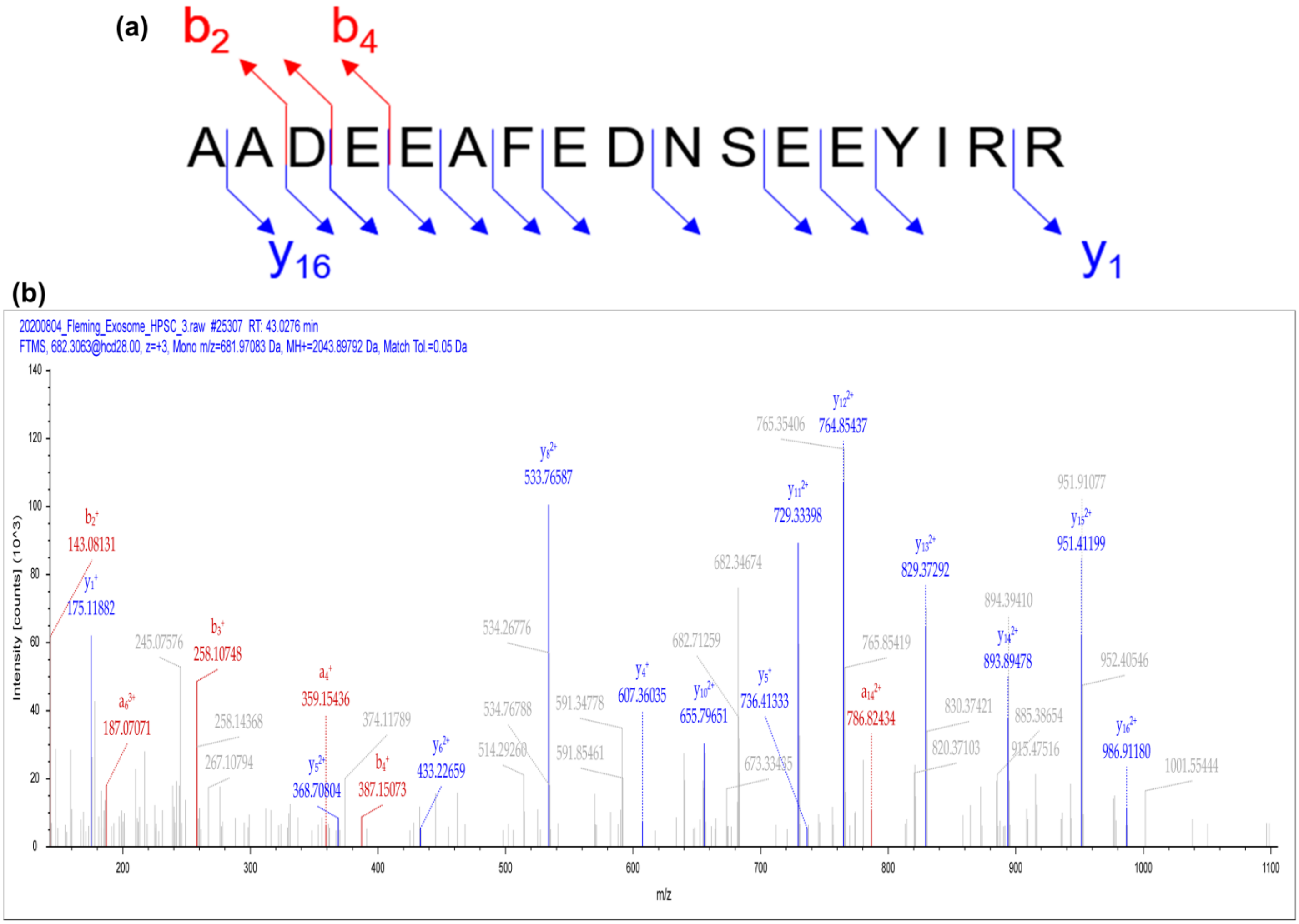

Fig. S13: **CSE1L, expressed in PDAC, is associated with poor survival**. The Kaplan-Meier survival plot used 174 PDAC patients identified from the Cancer Genome Atlas (59) with CSE1L gene expression and clinical outcome data. Patients were stratified by median CSE1L expression for survival analysis. We found that higher expression of CSE1L was associated with a significantly poorer survival outcome (log-rank p-value = 9.95E-04).

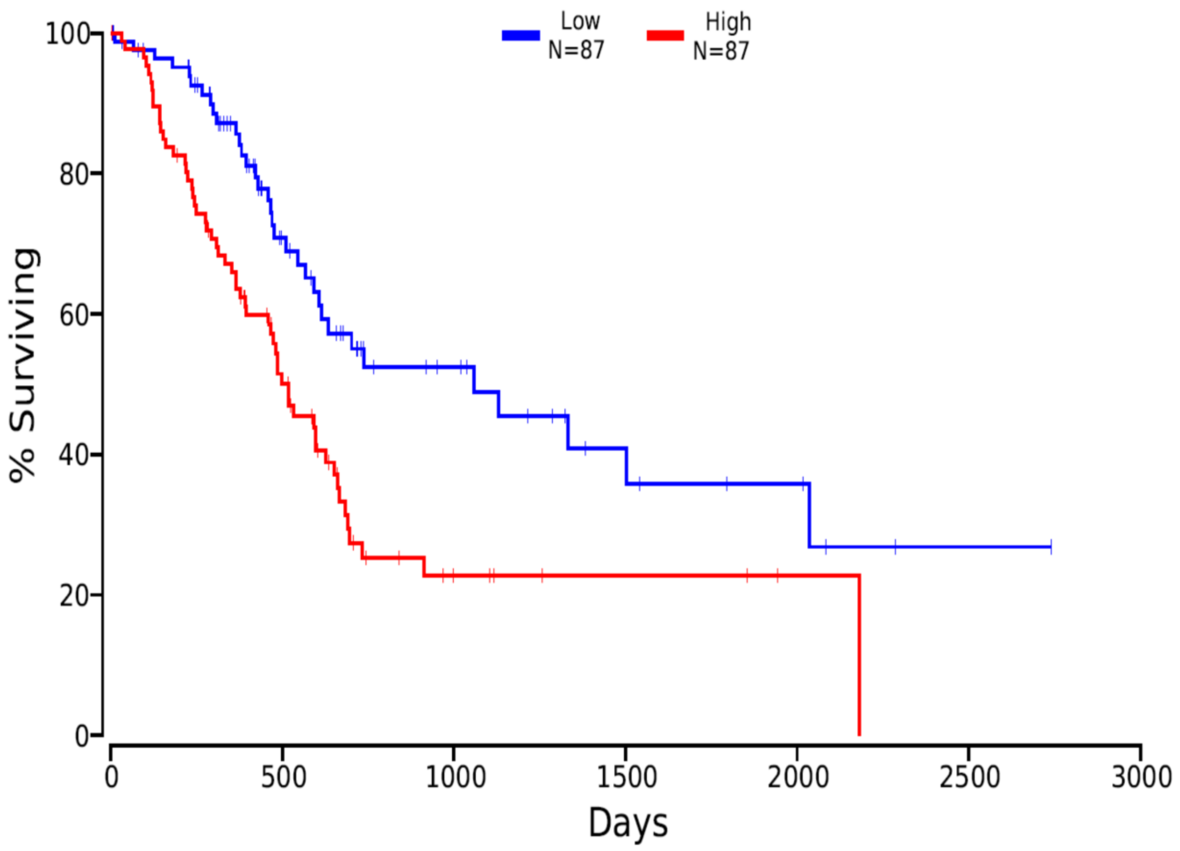

Supp video S1: Nanoparticle tracking analysis of small extracellular vesicles from PDAC associated human pancreatic stellate cells. This video presents a representative example of nanoparticle tracking analysis (NTA) of small extracellular vesicles (sEVs) from PDAC associated human pancreatic stellate cells (HPSC) grown in culture. NTA was performed using a NanoSight NS300 (Malvern Panalytical, Malvern, UK) equipped with a 488 nm blue laser module, flow-cell top-plate, integrated temperature control, and a single-syringe pump module. NTA3.4 software (Malvern) was used for video capture and analysis. In short, sEVs were isolated by differential ultracentrifugation from conditioned media (CM) from immortalized HPSC isolated from primary PDAC tumors. CM was harvested when cells reached 70-80% confluency and cleared of cells, cell debris, and large membrane vesicles by sequential centrifugation at 500 x g and 12,000 x g for 30 min each. The cleared CM was then filtered through 0.2µM filter unit. sEVs were then collected after centrifugation at 100,000 x g for 2h using SWT32i (Beckman) swinging buckets in Beckman Coulter ultracentrifuge. The sEVs were washed with PBS at 100,000 x g for 2 hrs. and the pellets were resuspended in 0.22 µM filtered 1x PBS. The concentration of particles and their size distributions were assessed by NTA performed in a NanoSight NS300 (Malvern Panalytical, Malvern, UK) equipped with a 488 nm blue laser module, flow-cell top-plate, integrated temperature control, and a single-syringe pump module. Samples were diluted using cell-culture grade water (Corning cat.# 25-005-CI) to produce a particle concentration in the range of 10^7–10^{10} particles/ml (as determined during an initial quick static measurement), which represents approximately 20–100 particles/frame (particles in the instrument's field of view). Final measurements consisted of 5 standard measurements of 1 min of duration each, at a controlled temperature of 25°C and under constant automatic flow (continuous syringe pump speed set to 50 arbitrary units). Camera level for video capture was set to 13 and detection threshold to 5 for all sample measurements. NTA 3.4 software (Malvern) was used for video capture and analysis.

Supplementary Tables 2 and 3 are both large Excel file datasets.

Supplementary Table 1: Parameter.text file from Maxquant

Supplementary Table 2: Uniprot membrane annotations

Supplementary Table 3: Proteomics analysis results of HPSC and HPaStec cells sEVs

Supplementary video: Nanoparticle tracking analysis of small extracellular vesicles from PDAC associated human pancreatic stellate cells.
