# Supplementary material for: A comparative Proteomics Analysis Identified Differentially Expressed Proteins in Pancreatic Cancer–Associated Stellate Cell Small Extracellular Vesicles
Source: Mol Cell Proteomics. 2022 Nov 2;21(12):100438. doi: 10.1016/j.mcpro.2022.100438 (PMC9792568; doi:10.1016/j.mcpro.2022.100438)
Supplement: Table S1 [file mmc3.docx]

**Supplementary Table S1: Parameter.text file from Maxquant.**

**Parameter Value**

**Version 1.5.2.8**

**User name msadmin**

**Machine name P16XWDB2**

**Date of writing 08/10/2020 19:39:25**

**Fixed modifications Carbamidomethyl (C)**

**Decoy mode revert**

**Special AAs KR**

**Include contaminants True**

**MS/MS tol. (FTMS) 20 ppm**

**Top MS/MS peaks per 100 Da. (FTMS) 12**

**MS/MS deisotoping (FTMS) True**

**MS/MS tol. (ITMS) 0.5 Da**

**Top MS/MS peaks per 100 Da. (ITMS) 8**

**MS/MS deisotoping (ITMS) False**

**MS/MS tol. (TOF) 40 ppm**

**Top MS/MS peaks per 100 Da. (TOF) 10**

**MS/MS deisotoping (TOF) True**

**MS/MS tol. (Unknown) 0.5 Da**

**Top MS/MS peaks per 100 Da. (Unknown) 8**

**MS/MS deisotoping (Unknown) False**

**PSM FDR 0.01**

**Protein FDR 0.01**

**Site FDR 0.01**

**Use Normalized Ratios For Occupancy True**

**Min. peptide Length 7**

**Min. score for unmodified peptides 0**

**Min. score for modified peptides 40**

**Min. delta score for unmodified peptides 0**

**Min. delta score for modified peptides 6**

**Min. unique peptides 0**

**Min. razor peptides 1**

**Min. peptides 1**

**Use only unmodified peptides and True**

**Modifications included in protein quantification Acetyl (Protein N-term);Oxidation (M)**

**Peptides used for protein quantification Razor**

**Discard unmodified counterpart peptides True**

**Min. ratio count 1**

**Re-quantify True**

**Use delta score False**

**iBAQ True**

**iBAQ log fit True**

**Match between runs True**

**Matching time window [min] 6**

**Alignment time window [min] 20**

**Find dependent peptides False**

**Fasta file C:\MaxQuant 1.5.2.8\Databases\SwissProt_HUMAN_2020_03.fasta**

**Labeled amino acid filtering True**

**Site tables Oxidation (M)Sites.txt**

**Decoy mode revert**

**Special AAs KR**

**Include contaminants True**

**RT shift False**

**Advanced ratios True**
